# Phospholipid-Coated Fe_3_O_4_ Nanoparticles Enable Rapid Screening of Putative Antimicrobial Peptides from *Clanis bilineata tsingtauica* Hemolymph

**DOI:** 10.3390/antibiotics15070702

**Published:** 2026-07-18

**Authors:** Zong-Nan Li, Lei Qian, Qing-Yi Li, Yi Qin, Pan Deng, Jian-Jun Guo, Huai-Jian Liao

**Affiliations:** 1 Institute of Leisure Agriculture, Jiangsu Academy of Agricultural Sciences, Nanjing 210014, China; 2 College of Biotechnology, Jiangsu University of Science and Technology, Zhenjiang 212100, China; 3 Provincial Key Laboratory for Agricultural Pest Management of Mountainous Region, Institute of Entomology, Guizhou University, Guiyang 550025, China

**Keywords:** *Clanis bilineata tsingtauica*, putative antimicrobial peptides, phospholipid coating, Fe_3_O_4_@L nanoparticles, de novo sequencing

## Abstract

**Background/Objectives**: Antimicrobial peptides (AMPs) in insect hemolymph are promising alternatives to conventional antibiotics, but their rapid screening from complex biological matrices remains difficult. This study aimed to develop a simple and efficient method for the enrichment and preliminary identification of putative AMPs from the hemolymph of *Clanis bilineata tsingtauica* larvae. **Methods**: Phospholipid-coated magnetic nanoparticles (Fe_3_O_4_@L) were prepared using membrane phospholipids identified from *Enterococcus faecium* by time-of-flight mass spectrometry. After nanoparticle characterization, enrichment conditions were optimized and the Fe_3_O_4_@L workflow was compared with a conventional ultrafiltration-electrophoresis-silver staining-mass spectrometry (UESM) method. Peptides were identified by de novo mass spectrometry, followed by sequence filtering, replicate screening, and in silico AMP prediction. Candidate peptides were chemically synthesized and evaluated for antibacterial activity, minimum inhibitory concentration (MIC), growth inhibition, morphological effects, and hemolytic activity. **Results**: Optimization of the Fe_3_O_4_@L method resulted in a 1.86-fold increase in peptide concentration in the enriched fraction. Compared with UESM, Fe_3_O_4_@L reduced the workflow from four steps to three and shortened processing time by 61.2%. De novo analysis generated 724 filtered peptides, of which 58 were reproducibly detected in at least two replicates, yielding five putative AMPs after activity prediction; no putative AMP was identified by UESM under the same conditions. Among the synthesized peptides, CBT-3 showed antibacterial activity against *E. faecium* and *Escherichia coli*, with MICs of 32 and 64 mg/L, respectively, and low hemolytic activity toward human erythrocytes. **Conclusions**: Fe_3_O_4_@L provides a simple and effective strategy for preliminary AMP screening from insect hemolymph, and CBT-3 represents a promising peptide candidate for further investigation.

## 1. Introduction

Antimicrobial peptides (AMPs) are small bioactive peptides found in biological tissues that exhibit antimicrobial activity [1]. They represent an important source of anti-infective agents and alternatives to antibiotics [2]. Furthermore, compared with conventional antibiotics, AMP-based products are generally considered less likely to raise concerns related to antibiotic residues [3]. Therefore, efficient identification of naturally occurring antimicrobial peptides is a prerequisite for the development of new antimicrobial drugs. Insects are the most abundant group of organisms and an important source of AMPs. In particular, the health benefits of certain edible insects are influenced by their endogenous AMPs [4]. *Clanis bilineata tsingtauica* is a distinctive edible insect native to Jiangsu, China. It has long been regarded as beneficial to health, particularly for nourishing the stomach. Its endogenous bioactive peptides may be promising for development in food, feed, and biopharmaceutical applications; however, research on its AMPs remains limited. Insect antimicrobial peptides display structural diversity (such as *α*-helixes and *β*-sheets), and these structural differences help explain why they use different antimicrobial mechanisms [5,6]. In addition to antimicrobial activity, hemolytic activity is a key parameter for assessing the application potential of insect-derived putative AMPs for use in medicine, food, animal husbandry, and related fields [7].

For naturally occurring AMPs, direct identification from biological materials typically involves isolation and purification. However, the complex composition of raw samples makes it difficult to separate antimicrobial components. Consequently, complex biological matrices require extensive pretreatment and multi-step purification, which increases processing time, causes sample loss, and adds operational complexity—ultimately reducing the overall efficiency of antimicrobial peptide isolation. For example, naturally occurring silkworm AMPs often require multiple extraction and chromatographic purification steps prior to mass spectrometric (MS) identification [8]. Moreover, the labor-intensive nature of multiple chromatographic separations likewise limits AMP recovery and separation efficiency [9]. Alternatively, putative AMPs can be screened from biological transcriptome data using bioinformatics analyses [10]. Nevertheless, computationally predicted candidates still require experimental verification to confirm their natural expression in the source organism [11]. Therefore, there is a need for simpler, more efficient strategies that leverage biologically meaningful interaction features to enrich naturally expressed putative AMPs from complex matrices and enable preliminary screening of these candidates.

Membrane binding is an early common feature of many antimicrobial peptides (AMPs). Most AMPs are positively charged and preferentially interact with negatively charged phospholipids on bacterial membranes through electrostatic attraction [5]. This initial membrane binding can trigger membrane rupture or facilitate peptide entry into the cell, ultimately leading to bacterial death. For example, the AMP Mop2 from *Moringa oleifera* seed hydrolysate caused irreversible membrane damage to *Staphylococcus aureus*, resulting in intracellular nucleotide release [12]. Other AMPs act on intracellular targets; however, they must first contact the bacterial plasma membrane via electrostatic interactions [13]. The AMP A11 inhibits *Acinetobacter baumannii* by disrupting intracellular processes, including energy metabolism, protein homeostasis, and fatty acid synthesis, and it enters the cell without disrupting the cytoplasmic membrane [14]. Therefore, bacterial membrane phospholipids may serve as an affinity-based basis for enriching and preliminarily screening membrane-affinitive AMPs. In our previous study, we isolated and identified a pathogenic bacterium, *Enterococcus faecium*, from infected pupae of *C. bilineata tsingtauica*. This strain exhibited strong pathogenicity toward *C. bilineata tsingtauica* larvae, providing a biologically relevant source of bacterial membrane phospholipids for the present study. Additionally, *E. faecium* membrane phospholipids provide a host-associated pathogen membrane environment that more closely recapitulates the pathogen interface encountered by the host’s innate immune defenses than do generic artificial lipid models. However, phospholipids alone are difficult to use directly as separation materials and therefore require a suitable carrier.

As carriers, nanoparticles offer several advantages, including a large specific surface area, high reactivity, biocompatibility, and stability [15]. Among them, magnetic nanoparticles are particularly attractive due to their facile surface functionalization, high specific surface area, compatibility with magnetic separation, and reusability [16]. Accordingly, magnetic nanoparticles are widely used in cell separation, protein purification, nucleic acid extraction, immunoprecipitation, and related applications [17]. Importantly, the functional performance of magnetic nanoparticles depends strongly on their surface coating materials [18]. If the coating of a nanocarrier can mimic the bacterial membrane environment, it may selectively adsorb candidate peptides that possess membrane affinity. Therefore, magnetic nanoparticles coated with plasma membrane phospholipids derived from a specific pathogenic bacterium can be used to enrich and initially screen membrane-interactive putative AMPs. In this way, a direct adsorption approach from biological tissues provides a simpler alternative for preliminary peptide enrichment, and the enriched fractions can then be subjected to MS identification.

In this study, we developed a pathogen-membrane-inspired magnetic enrichment strategy for the rapid pre-screening of putative AMPs from *C. bilineata tsingtauica* hemolymph. Specifically, the phospholipids were isolated and characterized from *E. faecium*, and phospholipid-coated Fe_3_O_4_ magnetic nanoparticles (Fe_3_O_4_@L) were prepared for peptide enrichment and preliminary screening. The enrichment conditions were then optimized by varying the nanoparticle dosage, adsorption time, elution time, pH, and temperature. Next, the Fe_3_O_4_@L-based workflow was compared with the conventional ultrafiltration-electrophoresis-silver staining-mass spectrometry (UESM) method in terms of the number of extraction steps, processing time, peptide recovery, and detection outcomes. Finally, peptides identified from the enriched fractions were chemically synthesized and assessed for antibacterial activity and hemolytic activity. Overall, this work provides a mechanism-based and operationally simple approach for the initial screening of putative AMPs from complex biological matrices.

## 2. Results

### 2.1. Identification of Fe_3_O_4_@L Coating Materials

**Figure 1:**
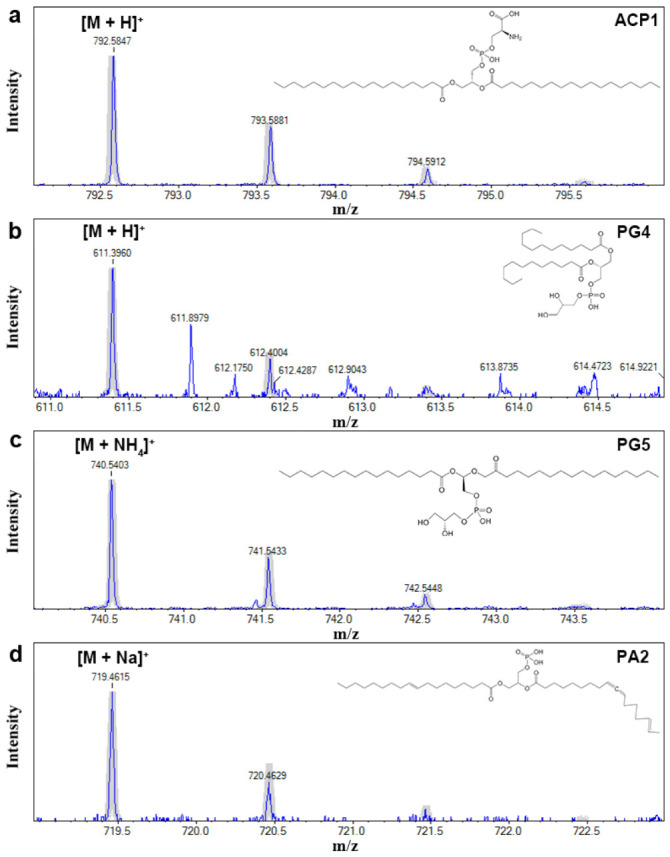
TOF-MS results of *E. faecium* cell membrane phospholipids: (**a**) ACP1, (**b**) PG4, (**c**) PG5 and (**d**) PA2.

Cell membrane phospholipids from *E. faecium*, known for their high lethality against *C. bilineata tsingtauica* larvae, were selected as the coating material for Fe_3_O_4_@L nanoparticles. The phospholipid composition of the extracted coating was characterized by TOF-MS (Figure S4). Candidate lipids were identified by comparing TOF-MS results with 24 phospholipids listed in the literature-based list of *E. faecium* phospholipids (Table S1), resulting in the detection of four candidate lipids, such as amino-containing phospholipid (ACP), lysyl-phosphatidylglycerol, phosphatidylglycerol (PG), cardiolipin, glycerolphospho-diglycodiacylglycerol (GP-GDGAG), and phosphatidic acid (PA). From the TOF-MS analyses, four phospholipids were tentatively identified (Table S2/Figure 1): ACP1 (C_42_H_82_NO_10_P), PG4 (C_30_H_59_O_10_P), PG5 (C_38_H_75_O_10_P) and PA2 (C_39_H_69_O_8_P). This coating strategy was designed to exploit electrostatic and hydrophobic interactions. Under acidic to near-neutral conditions, the positively charged Fe_3_O_4_ surface may facilitate adsorption of negatively charged phospholipid headgroups, while hydrophobic interactions among lipid tails likely contribute to stabilization of the coating layer.

### 2.2. Characterization of Fe_3_O_4_@L Nanoparticles

**Figure 2:**
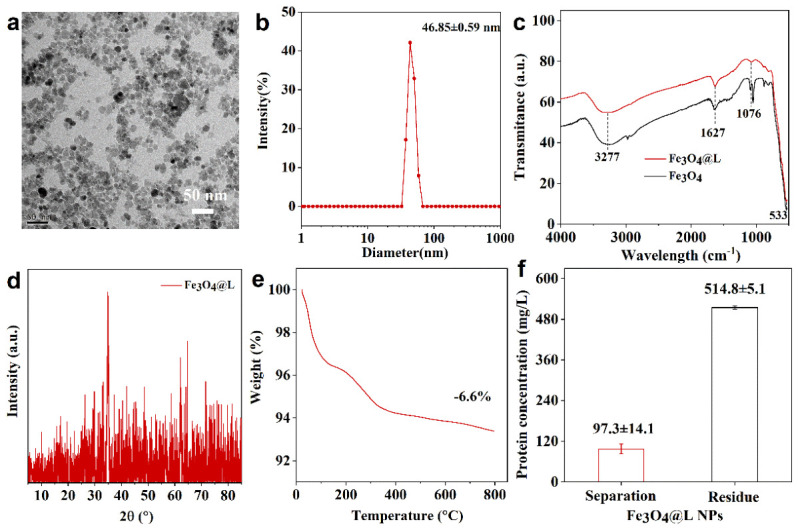
Characterization of Fe_3_O_4_@L nanoparticles using (**a**) transmission electron microscope (TEM), (**b**) dynamic light scattering (DLS), (**c**) Fourier transform infrared spectroscopy (FTIR), (**d**) X-ray diffraction (XRD), (**e**) thermogravimetric analysis (TGA), and (**f**) protein recovery. (**b**,**f**) show the particle size and adsorption capacity of Fe_3_O_4_@L nanoparticles in aqueous solution, while (**a**,**c**–**e**) present the properties of Fe_3_O_4_@L nanoparticle powder. Data are presented as means ± SD from three independent experiments. The scale bar in (**a**) represents 50 nm.

Fe_3_O_4_@L nanoparticles were synthesized via a one-pot coprecipitation method and collected as a fine black powder upon drying (Figure 2). TEM revealed irregularly shaped, aggregated nanoparticles, with individual domains measuring less than 50 nm (Figure 2a). DLS analysis determined a hydrodynamic diameter of 46.9 ± 0.6 nm (Figure 2b), confirming that the nanoparticles remained within the nanoscale range in aqueous suspension. FTIR spectra of bare Fe_3_O_4_ and Fe_3_O_4_@L are presented in Figure 2c. Both samples exhibited a pronounced absorption band at approximately 533 cm^−1^, characteristic of Fe-O vibration. Relative to bare Fe_3_O_4_, Fe_3_O_4_@L displayed additional or intensified bands at 3277, 1627, and 1076 cm^−1^, which are likely attributed to hydroxyl- and phosphate groups associated with the phospholipid coating, indicating successful surface modification. XRD analysis demonstrated that the crystalline structure of the iron oxide core was preserved following synthesis (Figure 2d). The absence of distinct diffraction peaks corresponding to the phospholipid layer is likely due to its low abundance and amorphous nature. TGA revealed a 6.6% mass loss between 20 and 800 °C (Figure 2e), consistent with the presence of a small quantity of organic material on the nanoparticle surface. The successful formation of the phospholipid coating was demonstrated by several lines of evidence: the emergence of lipid-associated signals in FTIR spectra, increased organic mass loss observed in TGA, and reduced diffraction peak intensity accompanied by elevated background noise in XRD analysis. To assess adsorption performance, the separation and recovery of peptides from hemolymph were evaluated by measuring peptide concentrations in the eluate and residual fractions (Figure 2f). The recovered peptide concentration in the eluate was 97.3 ± 14.1 mg/L, whereas 514.8 ± 5.1 mg/L remained in the residue, corresponding to an apparent recovery rate of 15.9% relative to the total measurable peptide content. Collectively, these results confirm the successful synthesis and functionalization of Fe_3_O_4_@L nanoparticles.

### 2.3. Optimization of Peptide Enrichment Using Fe_3_O_4_@L Nanoparticles

**Figure 3:**
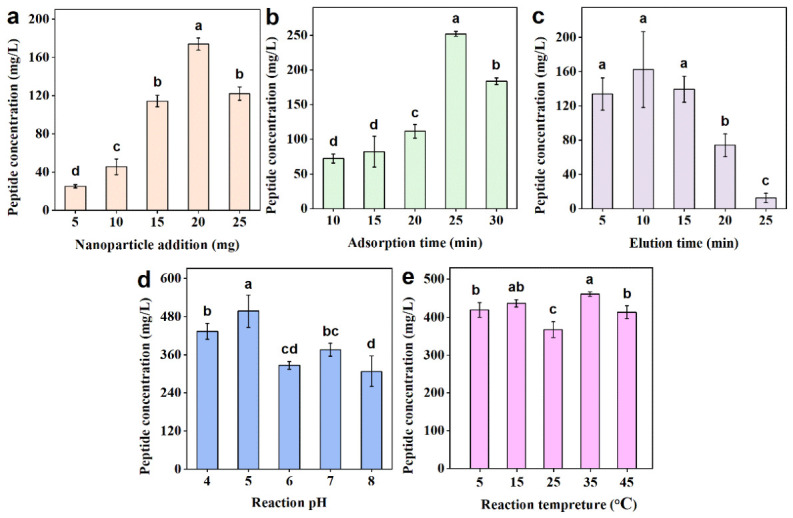
Peptide concentration in the eluate under different enrichment conditions: (**a**) nanoparticle addition, (**b**) adsorption time, (**c**) elution time, (**d**) reaction pH, and (**e**) reaction temperature. Data are presented as means ± SD from three independent replicates. Different lowercase letters indicate statistically significant differences (*p* < 0.05).

The peptide enrichment capability of Fe_3_O_4_@L nanoparticles was evaluated by measuring peptide concentration in the eluate under various conditions (Figure 3). Initially, the effect of nanoparticle dosage was examined (Figure 3a). Peptide concentrations in the eluate did not differ significantly when 5–15 mg of nanoparticles were added. At 20 mg, the peptide concentration increased markedly, reaching the highest average value of 173.7 ± 6.4 mg/L. Further increases in nanoparticle dosage did not significantly affect peptide concentration; therefore, 20 mg was selected as the optimal amount. Next, adsorption time was optimized (Figure 3b). No significant change in peptide concentration was observed between 10 and 15 min. A substantial increase occurred at 20 min, with the highest mean peptide concentration achieved at 25 min (251.9 ± 3.7 mg/L). Extending the adsorption time to 30 min resulted in a significant decrease in peptide recovery; thus, 25 min was chosen as the optimal adsorption time. The optimal elution time was subsequently investigated (Figure 3c). Peptide concentrations in the eluate were similar across 5–15 min, with a peak concentration at 10 min (162.4 ± 44.5 mg/L). However, peptide concentrations decreased significantly at elution times of 20–25 min. Consequently, an elution time of 10 min was selected. Reaction pH and temperature were further optimized. As pH increased from 4 to 5, peptide concentration in the eluate rose significantly, reaching 497.6 ± 50.7 mg/L (Figure 3d). Higher pH values resulted in decreased peptide concentrations; therefore, the optimal reaction pH was set to 5. Temperature optimization revealed that peptide concentration in the eluate increased with temperature and peaked at 35 °C (461.2 ± 6.1 mg/L), followed by a significant decrease at 45 °C (Figure 3e). Accordingly, the optimal reaction temperature was established at 35 °C. In the present study, optimization of Fe_3_O_4_@L enrichment conditions was primarily guided by total peptide concentration in the eluate, serving as an operational response indicator to enhance peptide recovery from the hemolymph matrix. However, we acknowledge that increased total peptide recovery does not necessarily reflect improved selectivity for AMPs. Consequently, the optimized conditions identified in this study should be regarded as those that enhance overall peptide enrichment efficiency for preliminary screening purposes, rather than conditions demonstrated to maximize AMP selectivity.

### 2.4. Reusability of Fe_3_O_4_@L Nanoparticles

The reusability of Fe_3_O_4_@L nanoparticles was evaluated through repeated adsorption-separation cycles, with peptide enrichment in larval hemolymph quantified after each cycle (Figure S5). The peptide concentration after the first use was 260.1 ± 15.3 mg/L. The second yielded a comparable concentration of 261.6 ± 6.3 mg/L, indicating that the phospholipid coating and magnetic recovery maintained performance initially. In subsequent cycles, enrichment efficiency gradually declined. The third cycle resulted in a moderate decrease to 231.5 ± 4.5 mg/L, while the fourth cycle exhibited a pronounced reduction to 69.1 ± 4.8 mg/L. The fifth run remained low at 67.4 ± 16.0 mg/L, corresponding to 25.8% of the maximum value observed.

### 2.5. Comparison of the Fe_3_O_4_@L Method with the UESM Method

**Figure 4:**
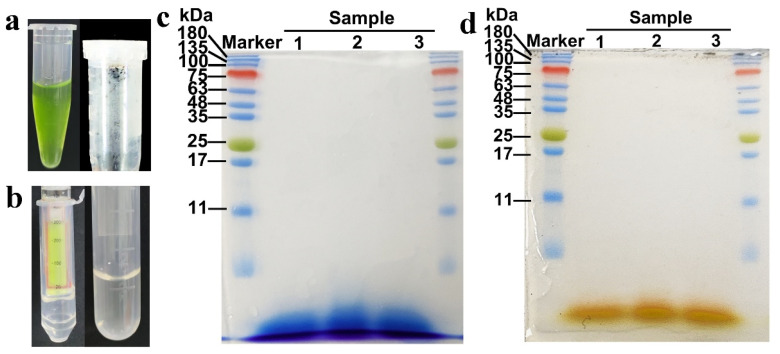
Comparison of the Fe_3_O_4_@L and UESM methods based on intermediate products: (**a**) peptide-enriched Fe_3_O_4_@L nanoparticles; (**b**) ultrafiltration filtrate of hemolymph; (**c**) SDS-PAGE gel image; and (**d**) silver-stained gel bands. Samples 1–3 represent peptides processed with UESM, and “marker” indicates the protein molecular weight marker.

To assess the efficiency of the Fe_3_O_4_@L method in separating and identifying putative AMPs, a comparative analysis was performed with the conventional UESM method (Table 1). The workflow analysis (Figure 4) revealed that Fe_3_O_4_@L comprises three steps: peptide adsorption (Figure 4a), elution, and de novo MS identification. In contrast, UESM involves four steps: ultrafiltration (Figure 4b), gel recovery via electrophoresis (Figure 4c), silver staining (Figure 4d), and subsequent MS identification. Experimental processing times, excluding pretreatment and intermediate steps, were also evaluated. For Fe_3_O_4_@L, magnetic particle recovery and elution each required 10 min, with a total processing time of approximately 165 min. In comparison, UESM required 30 min for gel preparation and 75 min for two-stage electrophoresis (see Supplementary Materials), while silver stain processing for MS compatibility took approximately 180 min, culminating in a total duration of about 425 min. The peptide concentrations obtained via Fe_3_O_4_@L was 97.3 mg/L, whereas UESM yielded 16 mg/mL. However, de novo MS analysis using Fe_3_O_4_@L produced 21,000–50,000 candidate peptide sequences per run, while UESM identified only 4–7 peptides. Notably, five putative AMPs were exclusively identified in the Fe_3_O_4_@L product; no putative AMPs were detected using UESM. Overall, for the isolation of putative AMPs from *C. bilineata tsingtauica* larvae, Fe_3_O_4_@L demonstrated substantial advantages over UESM in workflow simplicity, processing time, and the number of peptide candidates suitable for downstream AMP screening.

### 2.6. Identification of Putative AMPs

Putative AMPs enriched using Fe_3_O_4_@L were identified via MS with de novo sequencing. Three independent replicates yielded 2.8 × 10^4^, 2.1 × 10^4^, and 5.0 × 10^4^ peptide sequences, respectively. Peptides with an average local confidence ≥ 85% and tag length > 3 were filtered from the MS output, resulting in 329, 192, and 203 sequences across replicates, for a total of 724 peptides. Peptides detected in at least two of the three replicates were further retained, yielding 58 screened peptides. Prediction of antibacterial activity narrowed this pool to five putative AMPs. Table 2 presents the sequences and structural properties of the five putative AMPs: CBT-1(TDLQKLLR), CBT-2 (KVTGLFK), CBT-3 (ADKVAGK), CBT-4 (YEVGKLPK), and CBT-5 (YELLKK), with sequence lengths of 8, 7, 7, 8, and 6 amino acids, respectively. All peptides exhibited low to moderate predicted hydrophobicity. At pH 7, CBT-2 had a net charge of +2, while the others had a net charge of +1. Secondary structure predictions indicated CBT-1, CBT-3, CBT-4 and CBT-5 are *α*-helical, and CBT-2 is a *β*-sheet. The three-dimensional structures provide further visualization of their spatial conformations. Based on repeated screening and in silico antimicrobial predictions, these five sequences were selected as putative AMPs and subsequently synthesized for functional testing. Using the UESM method, 4, 5, and 7 sequences were identified in three separate runs. No sequences were detected consistently across all three runs; however, three sequences appeared in two rounds: SLPSEISADYINDTLTSNMSNKVTWELASSGAIGFSRK (rounds 1 and 2), QHLERVLMHALILWRSLPNAICEMNQR (rounds 1 and 2), and KKPNDR (rounds 2 and 3). Online prediction tools indicated that none of these repeatedly detected sequences, nor the other ten sequences identified by UESM, possessed antimicrobial potential.

### 2.7. Antibacterial Activity of Peptide-Enriched Fractions and Synthetic Putative AMPs

**Figure 5:**
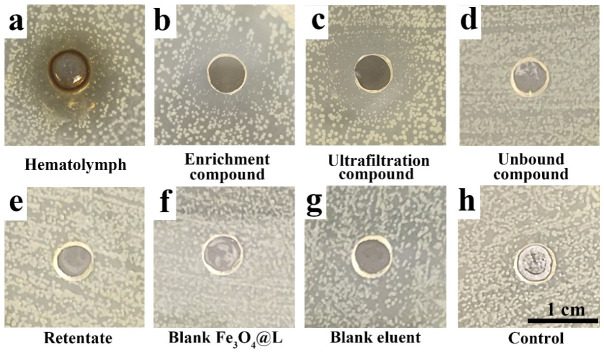
Inhibition zones against *E. faecium* produced by different fractions and controls: (**a**) hemolymph, (**b**) enrichment compound, (**c**) ultrafiltration compound, (**d**) unbound compound, (**e**) retentate, (**f**) blank Fe_3_O_4_@L, (**g**) blank eluent, and (**h**) negative control. The components in (**b**,**d**,**f**,**g**) were obtained using Fe_3_O_4_@L, while those in (**c**,**e**) were obtained using the UESM method. Scale bar = 1 cm.

The antibacterial activity of the peptide enriched fractions against *E. faecium* was evaluated using an inhibition zone assay (Figure 5). Hemolymph from *C. bilineata tsingtauica* larvae produced a distinct inhibition zone and darkened the surrounding culture medium (Figure 5a). Fractions obtained through both Fe_3_O_4_@L enrichment and UESM processing also generated inhibition zones (Figure 5b,c), whereas the unbound fraction and non-ultrafiltration fraction did not exhibit antimicrobial effects (Figure 5d,e). Additionally, blank Fe_3_O_4_@L nanoparticles, blank eluate, and negative controls showed no inhibition zones (Figure 5f–h).

**Figure 6:**
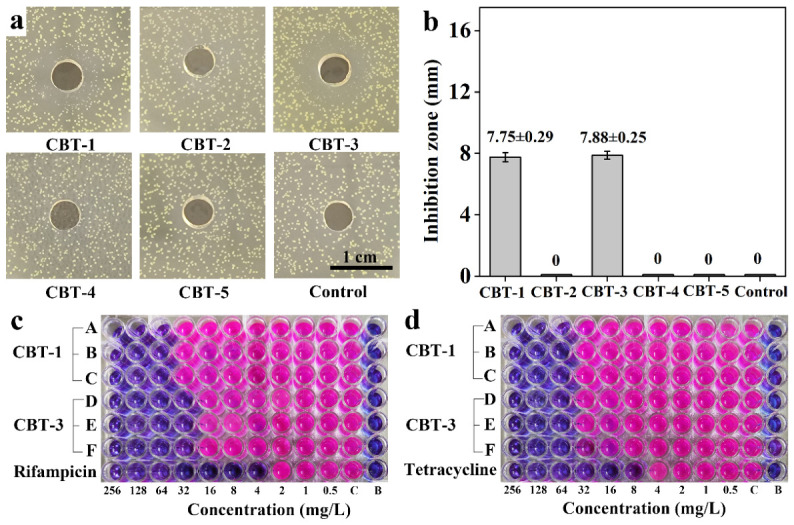
Antibacterial activity of synthetic putative AMPs. (**a**) Representative inhibition zones and (**b**) inhibition zone diameters. Antibacterial effects of CBT-1 and CBT-3 against (**c**) *E. faecium* and (**d**) *E. coli* were evaluated using resazurin-based 96-well MIC assays. Well diameter: 6 mm. Data are presented as means ± SD from three independent experiments. Rifampicin and tetracycline served as positive controls; C, bacterial control without peptide or antibiotic; B, blank control without bacteria.

Due to the low native yield of each putative AMP, five candidate peptides were synthesized via solid-phase peptide synthesis and subsequently evaluated for antimicrobial activity (Figure 6). The antibacterial activities of CBT-1 through CBT-5 against *E. faecium* were assessed by inhibition zone assay. Inhibition zones were observed only for CBT-1 and CBT-3 (Figure 6a), with mean diameters of 7.75 ± 0.29 mm and 7.88 ± 0.25 mm, respectively; other peptides showed no zones of inhibition (Figure 6b). Accordingly, CBT-1 and CBT-3 were validated as active AMPs under the test conditions. The MICs of CBT-1 and CBT-3 were then determined against both the representative Gram-positive bacterium *E. faecium* and the representative Gram-negative bacterium *E. coli*. For *E. faecium*, the MICs for CBT-1 and CBT-3 were 64 and 32 mg/L, respectively (Figure 6c). For *E. coli*, both peptides exhibited MICs of 64 mg/L (Figure 6d). As positive controls, rifampin and tetracycline demonstrated MICs of 4 mg/L and 8 mg/L against *E. faecium* and *E. coli*, respectively, indicating higher potency than the peptides. Given its lower MIC against *E. faecium*, CBT-3 was selected as the lead candidate for further study.

**Figure 7:**
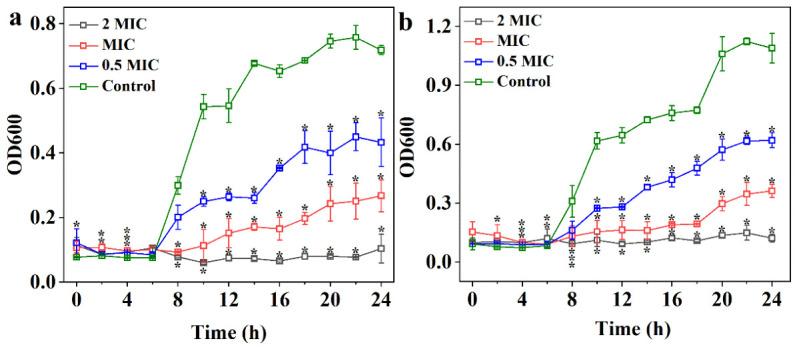
Growth inhibition curves of CBT-3 against (**a**) *E. faecium* and (**b**) *E. coli*. Asterisks indicate significant differences compared to the corresponding control group (*p* < 0.05). Data are presented as means ± SD from three independent replicates. MIC, minimum inhibitory concentration.

The antibacterial activity of CBT-3 against *E. faecium* and *E. coli* over 24 h was further evaluated using a growth inhibition assay at concentrations ranging from 0.5 to 2 × MIC (Figure 7). From 0 to 6 h, OD values in all groups remained low, consistent with the initial lag phase. From 8 to 24 h, however, OD values for all CBT-3 treatments were significantly lower than those of the control group (*p* < 0.05). For *E. faecium*, logarithmic growth began at 8 h, with inhibition rates for 2 × MIC, MIC, and 0.5 × MIC at 73.8%, 68.9%, and 32.9%, respectively (Figure 7a). At 12, 16, and 24 h, the inhibition rates for 2 × MIC were 86.4%, 90.0%, and 85.6%; for MIC, 72.2%, 74.7%, and 62.8%; and for 0.5 × MIC, 51.7%, 46.0%, and 39.7%. Similarly, for *E. coli*, logarithmic growth occurred at 8 h with inhibition rates of 69.9%, 57.5%, and 47.8% for 2 × MIC, MIC, and 0.5 × MIC, respectively (Figure 7b). At 12, 16, and 24 h, the inhibition rates for 2 × MIC were 85.8%, 83.9%, and 88.8%; for MIC, 74.7%, 74.9%, and 66.7%; and for 0.5 × MIC, 56.5%, 44.8%, and 43.0%.

**Figure 8:**
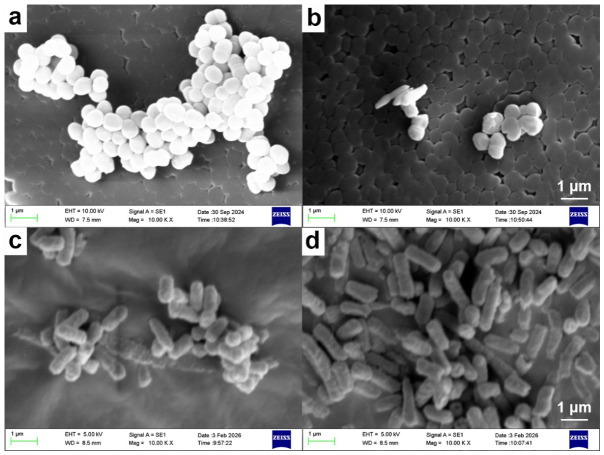
Scanning electron microscope (SEM) characterization of *E. faecium* and *E. coli* in the presence or absence of CBT-3. (**a**) untreated *E. faecium*; (**b**) CBT-3-treated *E. faecium*; (**c**) untreated *E. coli*; (**d**) CBT-3-treated *E. coli*. CBT-3 was applied at the MIC for 1 h. Scale bar = 1 μm.

The morphological effects of CBT-3 on *E. faecium* and *E. coli* at the MIC were examined by SEM (Figure 8). Untreated *E. faecium* displayed regular coccoid morphology with smooth, intact surfaces (Figure 8a). In contrast, CBT-3-treated cells exhibited irregular, elongated forms with rough and angular surfaces, although no overt structural breakage was observed (Figure 8b). For *E. coli*, untreated cells appeared as uniform, smooth rods with intact structures (Figure 8c). Following CBT-3 treatment, cells displayed distorted shapes, irregular contours, wrinkled surfaces, and localized collapse (Figure 8d). These findings suggest that CBT-3 disrupts the bacterial surface and induces marked morphological alterations.

### 2.8. Hemolytic Activity and Preliminary Biosafety Assessment

**Figure 9:**
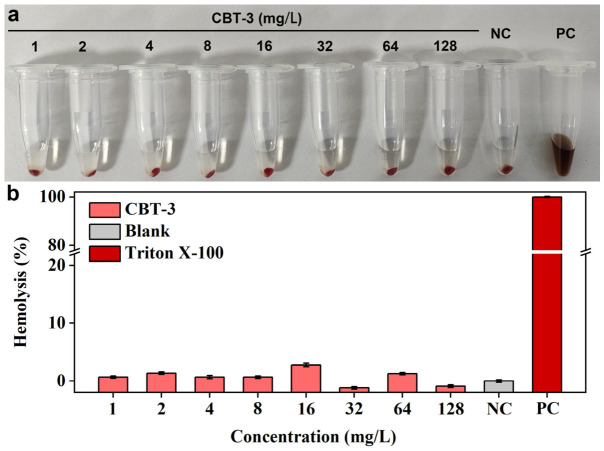
Hemolytic activity of CBT-3 toward human erythrocytes. (**a**) Representative appearance of erythrocyte suspensions after exposure to varying concentrations of CBT-3. (**b**) Quantitative hemolysis rates. Negative control (NC) contained no CBT-3; positive control (PC) contained 0.1% Triton X-100. Hemolysis data are shown as means ± SD from three independent experiments.

The preliminary biosafety of the AMP CBT-3 was evaluated by assessing its hemolytic activity on human erythrocytes (Figure 9). Supernatants obtained from erythrocytes treated with CBT-3 at concentrations ranging from 1 to 128 mg/L, as well as those from the negative control group, displayed no visible red coloration (Figure 9a). Quantitative analysis showed that the hemolysis rate in all CBT-3-treated groups remained below 3% (Figure 9b). Specifically, at the MICs determined for *E. faecium* (32 mg/L) and *E. coli* (64 mg/L), CBT-3 induced hemolysis rates of 0% and 1.2%, respectively. These results indicate that CBT-3 exhibits minimal hemolytic activity toward human erythrocytes under the tested conditions.

## 3. Discussion

The biological significance of Fe_3_O_4_@L in this study stems from its design, which was inspired by pathogen membranes and enables the selective enrichment of membrane-active peptide components from the complex hemolymph matrix of *C. bilineata tsingtauica*. Fe_3_O_4_@L itself is not an antimicrobial agent but serves as a biomimetic screening tool. By mimicking the surface of bacterial phospholipids, it facilitates the initial capture of putative antimicrobial peptides (AMPs) with affinity for bacterial membrane components. This approach is biologically important because many AMPs exert their effects through preferential interactions with microbial membranes. Additionally, the absence of inhibition zones in both the blank Fe_3_O_4_@L and the blank elution buffer controls confirms that the observed antimicrobial activity originates from the enriched peptide components rather than the nanoparticles themselves. Compared to traditional UESM workflows, Fe_3_O_4_@L reduces the number of processing steps and shortens screening times, demonstrating practical value for rapid prescreening of putative AMPs. Overall, Fe_3_O_4_@L is biologically significant not only for its biomimetic enrichment mechanism but also as a user-friendly platform for identifying bioactive peptide candidates from natural insect sources.

The Fe_3_O_4_@L platform is effective for AMP adsorption because it couples electrostatic attraction with hydrophobic insertion. In acidic/neutral conditions, negatively charged phospholipid headgroups on the Fe_3_O_4_@L surface bind positively charged AMPs; after initial electrostatic attachment, the amphiphilic AMPs insert their hydrophobic regions into the lipid tail domain of the coating. This “two-step” interaction is meant to mirror how AMPs recognize and engage real bacterial membranes, explaining why lipid-coated nanoparticles can capture AMPs that may not bind well to plain inorganic surfaces or generic synthetic lipids. To justify this biomimetic design, prior literature on *E. faecium* phospholipid composition was used as the basis for constructing a candidate lipid list. Studies reported that *E. faecium* phospholipids include lysyl-phosphatidylglycerol (LPG), phosphatidylglycerol (PG), cardiolipin, and GP-DGDAG [19], and additional work further noted mono-/dihexosyl diacylglycerols (MHDAG/DHDAG) and GPDD (glycerolphosphate diglucosyl diacylglycerol) [20]. In the present study, filtering the TOF-MS results with these reported lipid classes led to identification of four likely components (including ACP1 as a phosphatidylserine-like, negatively charged phospholipid) [21]. PG5 and PA are edible oil constituents [22]. By coating Fe_3_O_4_ with these bacterial membrane lipids, the nanoparticles function as “miniature bacteria”, providing a membrane-like chemical environment (lipid diversity, charge patterning, and amphiphilicity) that governs AMP binding. Ultimately, AMP adsorption is driven by matching between AMP amphiphilicity and the complementary electrostatic/hydrophobic features of the membrane-mimic coating, rather than by the nanoparticle core itself. Furthermore, the composition of bacterial membranes varies across species; thus, the current phospholipid coating does not uniformly represent all bacterial membranes. Consequently, the enrichment strategy employed in this study should be considered a species-dependent, membrane-mimicking screening platform, rather than a universal optimization system for all categories of AMPs. Future research should compare phospholipid or mixed membrane coatings derived from different bacterial species to further evaluate and enhance the broad applicability of this method.

The Fe_3_O_4_@L particles have an appropriate size, and TEM results indicate that the primary particle units are on the nanoscale. The size of the hydrated particles increases, primarily due to the synthesis conditions and the intrinsic material properties. For example, simply changing the alkali used in the coprecipitation step (e.g., replacing NH_4_OH with NaOH) can increase the Fe_3_O_4_ particle size by 22 nm [23]. Fe_3_O_4_@L nanoparticles are magnetic and may show some aggregation in aqueous suspension. FTIR analysis was used to compare bare Fe_3_O_4_ with phospholipid-coated Fe_3_O_4_@L. Both spectra showed a characteristic absorption band at approximately 533 cm^−1^, which is attributable to Fe-O stretching vibration [24]. In addition, Fe_3_O_4_@L exhibited changes in the bands around 3277, 1627, and 1076 cm^−1^ relative to bare Fe_3_O_4_. These bands may be associated with surface hydroxyl groups, adsorbed water, and phosphate-containing organic moieties derived from the phospholipid coating [25,26]. Together, these spectral differences suggest successful surface modification of Fe_3_O_4_ by phospholipids. XRD analysis further indicated that the nanoparticles retained a crystalline iron oxide core after coating. No distinct diffraction peaks attributable to the phospholipid layer were observed, likely because of its low content and limited crystallinity. TGA revealed a mass loss of 6.6% between 20 and 800 °C, which mainly reflects pyrolysis of the organic phospholipid layer on the nanoparticle surface. This loss is comparable to the reported mass fraction of quaternary ammonium-modified plant polyphenol coating [27], suggesting that the maximum mass percentage of the Fe_3_O_4_@L surface coating is effectively fixed. In this study, no surfactants were added during Fe_3_O_4_@L synthesis, which helps avoid introducing undesired impurities into the Fe_3_O_4_@L coating. Fe_3_O_4_@L nanoparticles were able to adsorb 15.9% of the total protein in larval hemolymph, demonstrating their adsorption capacity.

Nanoparticle functionalization provides a basis for peptide adsorption. Magnetic polymer microspheres (Fe_3_O_4_@PMAA@Ni) have been reported to adsorb histidine-rich proteins at capacities up to 2660 mg/g [28]. The lower adsorption capacity of Fe_3_O_4_@L may be attributed to differences in the phospholipid coating material. For example, 1 mL of RBC membrane-coated Fe_3_O_4_@RBC nanoparticles (containing 1 mg of iron) adsorbed 0.025–0.05 mg/mL of protein when mixed with 3 mL of mouse whole blood [29]. Likewise, this study achieved substantial peptide adsorption using a relatively low number of nanoparticles, highlighting the adsorption advantage conferred by a high specific surface area. Peptide adsorption and desorption behavior further reflected the interaction mechanism. Metal-chelated magnetic nanoparticles of purified protein C reached adsorption stability after 1 h [30]. In this study, Fe_3_O_4_@L nanoparticles exhibited faster adsorption kinetics, likely because electrostatic interactions directly promoted the adsorption of multiple positively charged peptides onto the coating. Peptide desorption is typically accomplished by adjusting the pH of the reaction environment. For instance, lysine desorption from cellulose nanofibers using magnetic TOCN-Fe_3_O_4_ nanoparticles required dispersion in an alkaline medium for 2 h [31]. In this study, desorption times were shorter and peptide recovery rates were higher under acidic conditions, indicating that the properties of the nanoparticles and adsorbed components influence desorption efficiency. Overall, the efficient separation of peptides relies on combining charge control with temperature control: pH adjustment can modulate electrostatic adsorption, whereas temperature primarily affects hydrophobic interactions [32]. In addition, future studies should include spiking experiments using positive and negative control peptides to more rigorously evaluate enrichment selectivity.

After several cycles of stable operation, the Fe_3_O_4_@L nanoparticles suddenly lost their coating functionality, commonly indicating irreversible fouling by peptides/biomolecules, incomplete desorption during regeneration, or loss/aggregation of the active surface, even if magnetic capture remains effective. The results of this study confirm that magnetic nanoparticles can endure multiple cycles and that the number of effective cycles can be increased through improved surface modification [33].

During the isolation of putative AMPs from the larvae of *C. bilineata tsingtauica*, the Fe_3_O_4_@L method showed clear advantages over the traditional UESM method, primarily in the following aspects. First, Fe_3_O_4_@L involves a simpler workflow. Compared with UESM, it imposes fewer requirements on equipment and operational complexity during peptide enrichment because functionalized magnetic nanoparticles reduce dependence on chromatography, centrifugation, precipitation, or ultrafiltration [34]. Second, the fast magnetic recovery speed of Fe_3_O_4_@L reduces the handling burden. Because nanoparticles are typically too small and readily dispersed, they are difficult to recover by centrifugation in solvents. By contrast, magnetic nanoparticles can be readily recovered by magnetic attraction, making them suitable nanocarriers [31]. Magnetic covalent organic framework particles can be fully recovered within 10 s [35], and each washing-recovery cycle for Fe_3_O_4_@L required no more than 1 min. Then formic acid elution in Fe_3_O_4_@L method is compatible with peptide recovery and MS. Improper AMP eluent will interfere with MS detection and lead to peptide decomposition. While the subsequent de novo sequencing of the Fe_3_O_4_@L method resolved a large number of peptide sequences in the sample [36]. In contrast, UESM is not suitable for low-abundance short peptides due to gel recovery/silver staining loss. Finally, Fe_3_O_4_@L better preserves antibacterial activity during enrichment.

De novo sequencing combined with bioinformatics analysis has been widely applied to optimize the identification of animal-derived peptides [37]. For example, 10,500 peptide sequences were obtained from the larval total RNA of *Psacothea hilaris* through de novo sequencing and analysis [36]. However, fewer peptides were identified in this study because, unlike whole-organism peptidomic analysis, the peptides analyzed here were derived from larval hemolymph and were further fractionated by Fe_3_O_4_@L. Moreover, hemolymph peptide concentrations in insects increase following infection with pathogenic bacteria; for instance, the hemolymph peptide concentration of *Hermetia illucens* increased to 0.739 μg/μL after infection with *E. coli* [38]. For *C. bilineata tsingtauica*, infection-triggered immune responses resulted in the secretion of numerous AMPs; accordingly, peptides with excessively low concentration values were excluded. To improve robustness, peptides detected in only one experiment were also removed to reduce the influence of individual experimental factors and enhance reproducibility. Finally, using the DBAASP database to predict peptide antimicrobial activity has improved screening efficiency and reduced downstream validation costs. After using DBAASP to predict antibacterial and hemolytic activities, one spider toxin peptide was identified from *Pardosa astrigera* transcriptome data as having antibacterial activity against *Pseudomonas aeruginosa* [39]. DBAASP performed similarly in this study, facilitating subsequent validation of the antibacterial activity of the target peptides. Future studies should integrate multiple AMP prediction algorithms for more robust candidate prioritization.

All five *C. bilineata tsingtauica* putative AMP sequences identified in this study contained fewer than 10 amino acids and were positively charged. This suggests that the negatively charged *E. faecium* phospholipid membrane coatings have a strong adsorption capacity for positively charged putative AMPs. The five sequences show physicochemical features partially resembling those of classical insect AMP families. CBT-1, CBT-3, CBT-4, and CBT-5 are short cationic peptides with predicted *α*-helical conformations, reminiscent of helical insect AMPs such as cecropins; CBT-2 contains glycine and may share physicochemical characteristics with gloverins [40]. However, given their very short length, they should be regarded as putative AMPs rather than definitive members of a classical AMP family. Notably, weak or no activity of some predicted peptides is not unusual for short natural putative AMPs. AMP Gh1 did not produce a significant inhibition zone against *E. faecalis* at the MIC [41].

Assessing whether the hemolymph and crude extracts of *C. bilineata tsingtauica* larvae exhibit antibacterial activity is an essential prerequisite for investigating the antibacterial properties of the resulting products. Accordingly, the antibacterial activity of all samples was initially screened using the inhibition zone assay. The inhibition zones observed for the hemolymph validate the reliability of the raw material. Both the Fe_3_O_4_@L and UESM crude extracts produced clear antibacterial zones, whereas the remaining products did not, indicating that both methods concentrated antibacterial components from the hemolymph. In contrast, neither the blank Fe_3_O_4_@L nanoparticles nor the eluate produced inhibition zones, ruling out any interference from these two factors on the antibacterial activity. The antibacterial activity of the Fe_3_O_4_@L fraction indicated that antibacterial components were retained during enrichment; however, peptide synthesis was required to verify the contributions of individual MS-identified candidates. Because the natural peptides recovered using the Fe_3_O_4_@L method were limited and comprised a heterogeneous mixture, antimicrobial and hemolytic assays were performed using five solid-phase-synthesized putative AMP.

*E. faecium* is clinically significant because of its multidrug resistance [42]. The MIC of the cationic peptide histrelin LL-37 against drug-resistant *E. faecium* was 72 mg/L but decreased to 36 mg/L in the synergistic presence of ampicillin (100 mg/L) [43]. In this study, the MICs of CBT-1 and CBT-3 against *E. faecium* were 64 mg/L and 32 mg/L, respectively, suggesting that CBT-3 has great application potential. The emergence of *E. faecium* resistance has been driven by antibiotic usage, promoting the directed evolution of a highly plastic genome toward drug resistance. Since the bacterial plasma membrane is highly conserved, it is difficult for bacteria to develop resistance to drugs that target this structure [44]. Thus, investigating plasma-membrane-targeting putative AMPs is important for suppressing drug resistance in *E. faecium*. Short cationic peptides may target conserved bacterial envelope components, making them a promising subject for further study. Here, CBT-3 showed antibacterial activity against both the representative Gram-positive bacterium *E. faecium* and the representative Gram-negative bacterium *E. coli*, indicating potential for pharmaceutical development. Putative AMPs intended for pharmaceutical use can be produced at scale via heterologous expression, enabled by efficient peptide secretion from microorganisms. For example, hidefensin-1, an AMP from the black soldier fly, was expressed in *E. coli*, yielding 13 mg of recombinant putative AMP per liter of culture [45]. Microbial heterologous expression therefore represents a feasible strategy for obtaining large quantities of CBT-3 in future work [46]. Expanding the antibacterial panel would be beneficial for more accurately delineating the activity spectrum and selectivity of the identified peptides. Nevertheless, the primary aim of the present study was to develop the peptide-enrichment strategy and to conduct initial functional validation of selected antimicrobial peptide candidates.

Antibacterial agents impair bacterial structural integrity. Halicin acts as a bacteriostatic agent against both *E. faecalis* and *E. faecium*. SEM analysis showed that treating *E. faecalis* with halicin at 20 mg/mL caused roughening of the bacterial cell surface, membrane deformation, and cell elongation [47]. SEM observations indicate that CBT-3 causes significant changes in the surface and morphology of both bacteria, consistent with impaired cell envelope integrity; however, the exact mechanism of action remains to be determined. Similarly, Pro10-1, an AMP designed based on beetle defensins, induced wrinkling of *E. coli* cells at the MIC; when the concentration was further increased, the cells progressively became shriveled, with marked cellular contraction [48]. Consistent with this pattern, CBT-3 also promoted surface roughening and contraction in *E. coli*. Similarly, CBT-3 appears to induce pronounced distortions in *E. coli*, including twisting, wrinkling, and local collapse, which may reflect an imbalance in intracellular tension and/or increased leakage of cellular contents. Overall, these results suggest that CBT-3 may cause ultrastructural damage to bacterial cells, leading to deformation.

The inhibition curve reflects the actual antibacterial activity of the AMP under aqueous conditions. Trematocine, an AMP derived from the Antarctic fish *Trematomus bernacchaii*, produced a bacteriostatic response against susceptible strains of *E. faecium* when cells were treated with 0.5–2 × MIC for 0–22 h; notably, no increase in OD values was observed over time at 2 × MIC [49]. Sarcotoxin 1C, an AMP isolated from the green bottle fly (*Lucilia sricata*), showed no increase in OD after 15 h when applied to *E. coli* at 0.185 μM, consistent with effective antibacterial activity [50]. Similar to the results above, CBT-3 showed no increase in OD values for *E. faecium* and *E. coli* at a concentration of 2 × MIC, indicating that the sustained inhibitory effect of CBT-3 in this study is concentration-dependent and is strongest at 2 × MIC.

Hemolysis is a key indicator for peptide safety assessment. The selective action of antimicrobial peptides is typically attributed to differences in membrane composition and surface charge between bacterial cells and mammalian cells. In this study, CBT-3 caused minimal hemolysis even at high concentrations, indicating low toxicity toward erythrocytes. These findings support further evaluation of CBT-3 for potential pharmaceutical and agricultural applications. It is worth noting that CBT-3 causes negligible hemolysis at its minimum inhibitory concentration (MIC), indicating that it is more selective for bacterial cells than for human red blood cells.

This study also has several limitations. A systematic investigation of the storage stability of Fe_3_O_4_@L was not conducted in this study. This aspect should be considered in future research focused on the functional optimization of Fe_3_O_4_@L. Also, owing to the limited yield of the Fe_3_O_4_@L-enriched product, the antimicrobial activity of the putative AMP natural products was not directly evaluated. In addition, this study primarily focused on the enrichment, identification, and preliminary validation of the antimicrobial activity of the peptides, as well as on evaluating selected properties of CBT-3. Comprehensive characterization of CBT-3, including its physicochemical properties and biological functions, remains to be thoroughly investigated. Future studies should assess its activity against a broader range of pathogens, conduct bactericidal kinetics analyses, and evaluate system stability. Furthermore, in this study, *E. faecium* and *E. coli* were chosen as representative Gram-positive and Gram-negative bacteria, respectively, for the initial evaluation of antibacterial activity. While other clinically important pathogens, such as *Staphylococcus aureus* and methicillin-resistant *S. aureus* (MRSA), are also highly relevant, they were not included in the current study. Future research should therefore investigate these organisms to more comprehensively assess the antibacterial spectrum of the identified peptides. Finally, the Fe_3_O_4_@L interface was constructed using phospholipids extracted from *E. faecium* and thus may preferentially enrich peptide candidates interacting with membrane components similar to those of this species. Given the known diversity in bacterial membrane composition, the current platform should not be considered universally representative of all bacterial targets. Additionally, because Fe_3_O_4_@L enrichment is driven by membrane-mimetic physicochemical interactions, non-antimicrobial membrane-binding peptides may also be retained. Furthermore, the optimization process was based on total peptide concentration rather than direct selectivity indicators for AMPs. Therefore, the Fe_3_O_4_@L approach is primarily intended for rapid screening of putative AMPs to assess peptide enrichment efficiency, rather than for their selective purification and detailed characterization, these studies can be conducted using complementary analytical methods. Future work should incorporate control peptide spike-in experiments, comparative analyses of recovery rates between antimicrobial and non-antimicrobial peptides, and alternative membrane models to systematically assess enrichment selectivity and generalizability.

## 4. Materials and Methods

### 4.1. Materials

Luria–Bertani (LB) broth medium and agar powder were obtained from Guangdong Huankai Microbial Technology Co., Ltd. (Zhaoqing, China). Methanol (CH_4_O), ferric chloride hexahydrate (FeCl_3_·6H_2_O), glycerol (C_3_H_8_O_3_), and sodium hydroxide (NaOH) were purchased from Sinopharm Chemical Reagent Co., Ltd. (Shanghai, China). Ferrous chloride tetrahydrate (FeCl_2_·4H_2_O) was obtained from Beijing Wokai Biotechnology Co., Ltd. (Beijing, China). Tert-butyl methyl ether (C_5_H_12_O) and formic acid (CH_2_O_2_, analytical grade) were purchased from Shanghai Aladdin Biochemical Technology Co., Ltd. (Shanghai, China).

### 4.2. Hemolymph Collection from C. bilineata tsingtauica Larvae

Both *C. bilineata tsingtauica* and soybean plants (*Glycine max* L., Ruidou-1) were reared at the Jiangsu Academy of Agricultural Sciences (Nanjing, Jiangsu, China). Larvae were placed on tender soybean leaves and maintained in plastic boxes in an insectary at 25 °C, 60% relative humidity, and a 14:10 h light-dark photoperiod. Hemolymph collection was performed following needle puncture to induce immune responses and AMP production (Figure S1) [51]. For each collection, the surface of 5th-instar larvae (Figure S1a) was disinfected with 70% ethanol. The larvae were then punctured with a needle dipped in a 10^9^ CFU/mL suspension of *E. faecium* (isolated from infected *C. bilineata tsingtauica* pupae; Figure S1b). After 24 h of incubation, the larval body surface was again swabbed with 70% ethanol. The larvae were then anesthetized on ice for 10 min on a clean bench, and hemolymph was collected by excising a proleg (Figure S1d). Collected hemolymph was centrifuged at 11,000 rpm for 15 min at 4 °C; the supernatant was then immediately frozen in liquid nitrogen and stored at −80 °C until further use.

### 4.3. Preparation and Characterization of Fe_3_O_4_@L Nanoparticles

Fe_3_O_4_ magnetic nanoparticles encapsulated in a phospholipid layer derived from the *E. faecium* plasma membrane (Fe_3_O_4_@L) were prepared for the isolation of putative AMPs (Figure S2). Phospholipids were extracted and characterized from *E. faecium* using a biphasic extraction method [52]. Briefly, a 0.1% (*v*/*v*) suspension of *E. faecium* at 10^9^ CFU/mL was inoculated into 1 L LB medium and cultured in a shaking incubator at 35 °C and 120 rpm for 2 d. Cells were harvested by centrifugation at 8000 rpm (Figure S2a), and the bacterial pellet was ground and resuspended by sequential addition of 1 mL methanol and 5.5 mL methyl tert-butyl ether, followed by 1 mL water and incubation for 20 min (Figure S2b). The upper (organic) phase was collected, and the lower phase was re-extracted with 6.5 mL of the same solvent mixture. The combined upper phases were transferred to clean centrifuge tubes (Figure S2c) and dried in a fume hood until the organic solvent was completely evaporated. The resulting phospholipids were dissolved in 1 mL HPLC-grade methanol (Figure S2d) and characterized using time-of-flight mass spectrometry (TOF-MS; AB SCIEX, TripleTOFTM 5600, Framingham, MA, USA).

Fe_3_O_4_@L nanoparticles were synthesized using a modified one-pot co-precipitation method (Figure S1c) [53]. Specifically, 2.31 g FeCl_3_·6H_2_O was dissolved in 7.5 mL water and filtered, while 1 g FeCl_2_·4H_2_O was dissolved in 6 mL water and similarly filtered. The two solutions were combined in a 500 mL beaker, diluted with 300 mL water, and stirred at 450 rpm for 20 min. The stirring rate was then increased to 1200 rpm, and an 8 g/L NaOH solution (approximately 110 mL) was added dropwise to adjust the pH above 10. After stirring for another 15 min, 2.5 mL of a 0.84 mg/mL phospholipid solution in methanol and 20 mL methanol were sequentially added, and the reaction was allowed to proceed for 20 min. The pH was then adjusted to 7 with dilute hydrochloric acid, followed by an additional 10 min of incubation. The resulting Fe_3_O_4_@L nanoparticles were magnetically separated, washed three times with distilled water, and dried at 60 °C.

The morphology of Fe_3_O_4_@L nanoparticles was examined by transmission electron microscopy (TEM; FEI, Tecnai G2F20, Hillsboro, OR, USA) after demagnetization and sonication. The particle size was measured by dynamic light scattering (DLS; Malvern, Zetasizer Nano ZSE, Malvern, UK). Functional groups were analyzed by Fourier transform infrared spectroscopy (FTIR; Thermo Scientific, Nicolet iS50, Madison, WI, USA), and the crystalline structure was examined by X-ray diffraction (XRD; Bruker AXS, D2 Phaser, Karlsruhe, Germany). Thermogravimetric analysis (TGA; Hitachi, TG/DTA7200, Tokyo, Japan) was also performed. Fe_3_O_4_@L nanoparticles were prepared independently in three batches using the same protocol, and comparable particle size distribution and FTIR profiles were obtained, indicating acceptable batch-to-batch reproducibility. To evaluate adsorption ability, the protein concentration in the hemolymph of *C. bilineata tsingtauica* larvae was measured at an optical density (OD) of 590 nm before and after particle adsorption using a spectrophotometer (Metash, UV-5200PC, Shanghai, China) and a Coomassie Brilliant Blue kit (Jiancheng, A045-2, Nanjing, China).

### 4.4. Optimization of the Fe_3_O_4_@L Method Conditions

The conditions for the Fe_3_O_4_@L method were systematically optimized to enhance peptide adsorption from the hemolymph of *C. bilineata tsingtauica*. Various amounts of Fe_3_O_4_@L nanoparticles (5, 10, 15, 20, and 25 mg) were added to 1.5 mL of hemolymph and incubated for 20 min. Following incubation, the nanoparticles were magnetically collected by holding them against the tube wall for 10 s, and the hemolymph was removed. The nanoparticles were then rinsed ten times with ultrapure water to remove blackening reaction byproducts and any unabsorbed materials. Elution was performed by adding 1.5 mL of 0.1% (*v*/*v*) formic acid to each tube, followed by incubation on a magnetic stirrer for 30 min to disrupt electrostatic interactions and release the adsorbed peptides. The peptide concentration in the eluent was determined, and the nanoparticle dosage yielding the highest peptide concentration was selected as optimal. To further optimize conditions, adsorption time was varied (10, 15, 20, 25, and 30 min) while keeping other parameters constant. Similarly, elution times were tested at 5, 10, 15, 20, and 25 min. The effects of reaction pH (4–8) and temperature (5–45 °C) were also evaluated. The reusability of Fe_3_O_4_@L nanoparticles was assessed by recovering and reusing the particles for four additional extraction cycles; measuring peptide enrichment in each cycle. A peptide standard curve (Figure S3) was constructed using CBT-3 standards at 5, 10, 25, 50, and 100 mg/L. Sample aliquots were added to a 96-well UV-transparent plate (Labselect, 12599, Beijing, China), and the absorbance at 214 nm was measured using a microplate reader (Spark Tecan, Männedorf, Switzerland) [54]. Sample peptide concentrations were then calculated from the standard curve. Each optimization experiment was performed using three independent replicate samples, and the peptide concentration data are presented as means ± SD (n = 3).

### 4.5. Isolation and Identification of Putative AMPs

Under optimized conditions, the eluate containing the enriched peptides was collected and filtered through a 0.22 μm membrane prior to analysis. Because the peptide discovery workflow was aimed at identifying potentially novel hemolymph peptides, a database-independent de novo sequencing strategy (Thermo Scientific, Fusion Lumos, Waltham, MA, USA; Xiamen Lvshengheng Biotechnology Co., Ltd., Xiamen, China) was adopted, rather than relying solely on database matching. De novo sequencing was performed using PEAKS Studio 8.5 (Bioinformatics Solutions Inc., Waterloo, ON, Canada). A conventional database-search-based false discovery rate (FDR) metric was not applied in the present study because peptide identification was performed using a de novo sequencing workflow intended for potentially novel peptide discovery. An Average Local Confidence (ALC) threshold of ≥85% was selected as a stringent empirical cutoff commonly used to improve confidence in short-peptide de novo assignments. To improve sequence reliability, stringent filtering criteria were used, including high-confidence sequence assignment, mass accuracy filtering, and reproducibility across independent replicates. LC–MS/MS analysis was performed on three independently prepared enriched hemolymph samples. Only peptides detected in at least two of the three independent replicates were retained. The antibacterial potential of the screened peptides was predicted using the linear AMP prediction tool provided by the DBAASP database (https://dbaasp.org/tools?page=linear-amp-prediction, accessed on 30 May 2026). In this study, the DBAASP output was used as a preliminary in silico prioritization step to identify peptide sequences with predicted AMP-like characteristics for subsequent synthesis and biological validation. Peptides predicted as putative AMPs by this platform, together with reproducible detection across independent MS replicates, were retained as candidate sequences for further consideration. No ensemble strategy combining multiple AMP prediction algorithms was applied in the current study; therefore, conflicting outputs among different predictors were not encountered. Based on the sequences of the putative AMPs, chemical formulas were generated using ChemDraw 20, and their secondary structures were predicted using the Chou-Fasman method via the ProteinIQ online tool (https://proteiniq.io/app/chou-fasman, accessed on 30 May 2026).

Additionally, a combination workflow of ultrafiltration, electrophoresis, silver staining, and mass spectrometry (UESM) was employed to isolate putative AMPs from the hemolymph of *C. bilineata tsingtauica* for comparative analysis with the Fe_3_O_4_@L separation method [55]. Hemolymph samples were fractionated using 10 kDa ultrafiltration tubes (Cobetter, 0.5 mL, Hangzhou, China). Each tube was reused for up to 2 mL of sample per run, with centrifugation at 4000 rpm for 5 min. The concentration of the obtained filtrate was determined, after which it was diluted with 1× phosphate-buffered saline (PBS) to a final peptide concentration of 10 mg/mL. Tricine-SDS-PAGE was performed as described in the Supplementary Materials. For each lane, 16 μL of sample was mixed with 4 μL of 5 × SDS-PAGE loading buffer (Phygene, PH0333, Fuzhou, China), heated at 100 °C for 3 min, cooled on ice, and loaded (20 μL per well). Electrophoresis was conducted on ice at 50 V for 15 min, followed by 90 V for 60 min. Peptide bands were visualized using a rapid silver staining kit (Phygene, PH1400, China). Selected bands were excised and submitted to Sangon Biotech (Shanghai) Co., Ltd. (Shanghai, China) for peptide identification by MS, and the antimicrobial activity of the identified peptides was subsequently predicted in silico using online tools.

### 4.6. Validation of Antimicrobial Activity of Synthetic Putative AMPs

Due to insufficient concentrations of naturally occurring peptide components for systematic antimicrobial testing, five putative AMP sequences were chemically synthesized for further validation. The putative AMPs were synthesized by solid-phase peptide synthesis (commissioned from Sangon Biotech [Shanghai] Co., Ltd., Shanghai, China) with a minimum purity of 90% and were subsequently desalted by the manufacturer. For the bacterial inhibition zone assay, *E. faecium* was cultured in LB medium for 24 h and diluted 2500-fold to approximately 2.5 × 10^6^ CFU/mL. Agar (5 g/L) was prepared in LB (20 g/L), sterilized at 121 °C, poured into plates, and allowed to solidify. Bacterial suspensions were evenly spread using a sterile swab. Four wells (6 mm diameter) were punched into each plate, and each sample was tested in four technical replicates. To compare the antimicrobial activity of the peptide-enriched fractions, 50 μL of each sample (larval hemolymph, enriched eluate, unbound fraction, blank nanoparticles, or blank eluate) was added to individual wells. Blank nanoparticles refer to nanoparticles that have not been exposed to hemolymph, while blank eluent refers to 0.1% formic acid that has not been mixed with nanoparticles. Regarding synthetic AMP, 50 μL of synthetic AMP solution (100 mg/L) was added to each well, while the control group received no sample. Plates were incubated at 37 °C for 24 h, after which inhibition zone diameters were measured.

The minimum inhibitory concentration (MIC) assay was performed as described by Zeng et al., with minor modifications [56]. Suspensions of *E. faecium* and *E. coli* (each at 5 × 10^5^ CFU/mL) were dispensed into two sterile 96-well plates (100 μL per well in A-G rows, except for the final well of each row). Additionally, 50 μL of sterile LB medium was added to each well in A-G rows. Next, 50 μL of synthetic AMP (1024 mg/L) was added to the first well of each row. CBT-1 was added to wells A_1_-C_1_, CBT-3 to wells D_1_-F_1_, and a positive control (rifampicin for *E. faecium*, tetracycline for *E. coli*, both at 1024 mg/L) to well G1. Serial twofold dilutions were performed across rows: 50 μL was transferred from well A_1_ to A_2_, mixed, then from A_2_ to well A_3_, continuing through A_10_, after which 50 μL from A_10_ was discarded. This process was repeated for each row. Plates were incubated at 37 °C for 8 h (contain *E. faecium*) and 16 h (contain *E. coli*). Following incubation, 30 μL of 0.015% resazurin was added to each well, and plates were incubated for an additional hour. MIC values were determined based on color change: pink indicated bacterial growth, while blue indicated inhibition.

Time-kill curves for the synthetic AMPs at various concentrations were then established. LB medium was prepared as a 20 g/L solution, sterilized by autoclaving at 121 °C for 20 min, and cooled. In a clean bench (SujingAntai, SW-CJ-1FD, Suzhou, China), 100 μL *E. faecium* and *E. coli* suspensions (10^9^ CFU/mL) were inoculated into 12 culture flasks. CBT-3 was added at concentrations corresponding to 2× MIC, 1× MIC, and 0.5× MIC for treatment groups; a single untreated control group was also included, with three replicates per group. Cultures were incubated in a shaking incubator (Nanjing Baisihe, ZQZY-90N, Nanjing, China) at 37 °C and 180 rpm for 24 h. At 2 h intervals, 3 mL samples were collected from each flask, and OD600 values were measured with a spectrophotometer. The bacterial growth inhibition rate at each time point was calculated as (OD_control_ − OD_treatment_)/OD_control_ × 100%.

### 4.7. Scanning Electron Microscopy

Scanning electron microscopy (SEM) was employed to investigate the effects of the putative AMP CBT-3 on the morphology. *E. faecium* and *E. coli*. Each bacterial culture was adjusted to 10^6^ CFU/mL and treated with CBT-3 at its respective MICs (36 mg/L for *E. faecium* and 64 mg/L for *E. coli*) for 1 h at 4 °C. Untreated cultures served as negative controls. Following treatment, samples were fixed and processed using an identical protocol for both strains. Briefly, cells were fixed with 2.5% (*v*/*v*) glutaraldehyde for 12 h at 4 °C and washed with PBS (0.1 M, pH 7.2). After washing, samples were dehydrated through a graded ethanol series (30%, 50%, 70%, 80%, 90%, and 100%), with each step lasting 10 min. The samples were then dried on a heated platform at 35 °C for 6 h and sputter-coated with gold. SEM imaging was conducted using a Zeiss, EVO-LS10instrument (Oberkochen, Germany).

### 4.8. Hemolytic Test

The hemolysis assay was performed based on the method described by Sim et al., with minor modifications [57]. Human red blood cells were washed three times with PBS. For the assay, 50 μL of washed red blood cells were mixed with CBT-3 at final concentrations of 1, 2, 4, 8, 16, 32, 64, and 128 mg/L in 1.5 mL centrifuge tubes, and PBS was added to a final volume of 200 μL. Negative controls contained only PBS, while positive controls included 0.1% Triton X-100 in place of the peptide. All samples were incubated at 37 °C for 1 h. Hemolysis was assessed both visually and by measuring absorbance at 540 nm. The hemolysis rate was calculated as follows: (A_sample_ − A_negative_)/(A_positive_ − A_negative_) × 100%, where A represents the OD540 values. Hemolysis assays were carried out in three independent replicate experiments. 

### 4.9. Statistical Analysis

For statistical analysis, the Shapiro–Wilk (S–W) test was first used to assess data normality. The Kruskal–Wallis test was applied to non-normally distributed data. For data meeting normality assumptions, homogeneity of variances was evaluated; if variances were homogeneous, one-way ANOVA followed by Tukey’s HSD post hoc test was used, while Tamhane’s T2 test was applied when variances were unequal. Statistically significant differences, as determined by the appropriate test, are indicated by different letters.

## 5. Conclusions

The Fe_3_O_4_@L method developed in this study enables efficient and rapid screening of putative AMPs from the hemolymph of *C. bilineata tsingtauica* larvae. The method offers operational simplicity, ease of magnetic particle recovery, and a streamlined enrichment workflow, making it well suited for preliminary AMP identification. Through de novo sequencing and in silico prediction, five putative AMP sequences were identified. Among these, CBT-3 demonstrated antibacterial activity against both *E. faecium* and *E. coli*, induced morphological changes in bacterial cells, and exhibited low hemolytic activity toward human erythrocytes under experimental conditions. Collectively, these findings confirm the utility of the Fe_3_O_4_@L method for AMP discovery and suggest CBT-3 as a promising candidate for further investigation. Nevertheless, additional studies are necessary to elucidate the antibacterial mechanism, evaluate broader bioactivity, and assess the in vivo safety of CBT-3. Further experimental validation of the remaining putative AMPs is also warranted.

## Figures and Tables

**Table 1:** Comparison of the Fe_3_O_4_@L method and the UESM method.

Indicators	Fe_3_O_4_@L	UESM
Instructions	Adsorption-Elution-MS	ultrafiltration–gel recovery by electrophoresis–silver staining–MS
Time spent	~165 min (Adsorption: 25 min; Magnetic particle recovery and elution: 20 min; Mass spectrometry: 120 min)	~425 min (Ultrafiltration: 20 min; gel electrophoresis: 105 min; silver staining: 90 min; MS 210 min (gel strip digestion: 180 min; MS/MS: 30 min))
Antibacterial activity of enriched components	Yes	Yes
Recovered peptide concentration	97.3 mg/L	16.0 mg/mL
Number of peptides sequences detected by MS	21,000–50,000	4–7
Number of putative peptides identified	5	0

UESM: ultrafiltration–gel recovery by electrophoresis–silver staining–mass spectrometry; MS: mass spectrometric.

**Table 2:** Chemical formula and structural information of putative AMPs from *C. bilineata tsingtauica*.

Putative AMP	Sequence	Structural Formula	Secondary Structure	Three-Dimensional Structure
CBT-1	TDLQKLLR	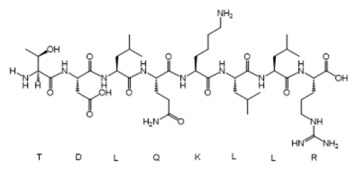	*α*-helix	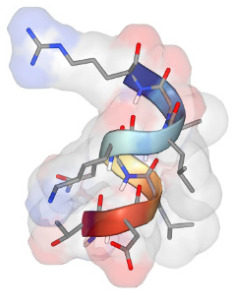
CBT-2	KVTGLFK	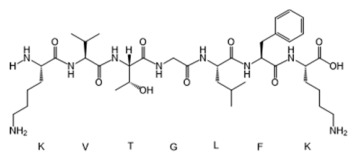	*β*-sheet	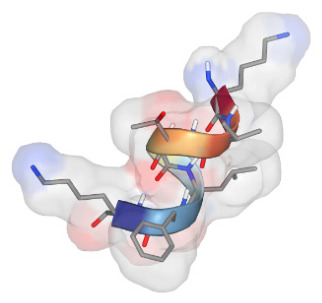
CBT-3	ADKVAGK	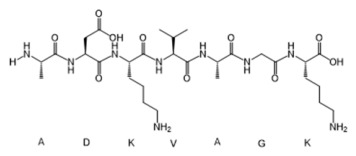	*α*-helix	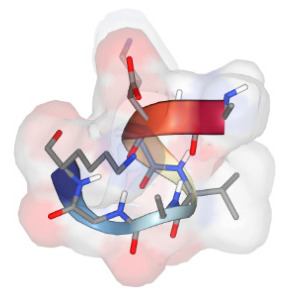
CBT-4	YEVGKLPK	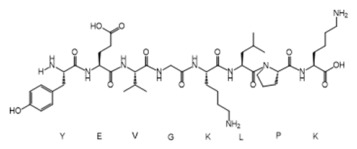	*α*-helix	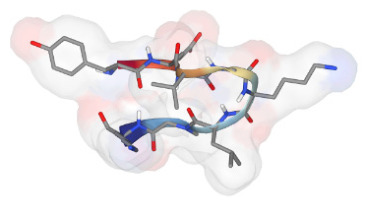
CBT-5	YELLKK	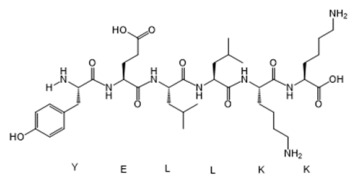	*α*-helix	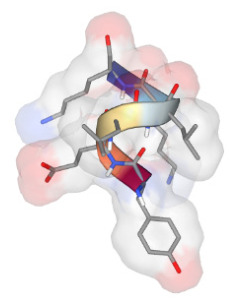

## Data Availability

Data will be made available on reasonable request.

## Supplementary Materials

The following supporting information can be downloaded at: https://www.mdpi.com/article/10.3390/antibiotics15070702/s1, Figure S1: Experimental materials: (a) 5th instar larvae and (b) pupae of *C. bilineata tsingtauica*; (c) Fe_3_O_4_ nanoparticles synthesized by co-precipitation; (d) larval hemolymph; (e) Fe_3_O_4_@L nanoparticles with adsorbed putative AMPs. The leftmost larva was alive; the two on the right were dead and blackened. Both pupae were also dead, blackened, and decomposed; Figure S2: Materials for phospholipid extraction from *E. faecium* plasma membrane using the aqueous two-phase system: (a) *E. faecium* precipitate, (b) two-phase system, (c) organic phase containing phospholipids, (d) phospholipids re-dissolved in methanol; Figure S3: Standard curve for antimicrobial peptide concentration; Figure S4: MALDI-TOF results of *E. faecium* membrane phospholipid analysis. Figure S5: Effect of Fe_3_O_4_@L nanoparticle reuse on enrichment of hemolymph peptides from *C. bilineata tsingtauica* larvae. Different lowercase letters indicate significant differences (*p* < 0.05); Table S1: Phospholipid detection list; Table S2: TOF-MS results for *E. faecium* phospholipid samples.

## Abbreviations

The following abbreviations are used in this manuscript:

AMP	Antimicrobial peptide
Fe_3_O_4_@L	Phospholipid-coated magnetic nanoparticles
UESM	Ultrafiltration-electrophoresis-silver staining-mass spectrometry
TOF-MS	Time-of-flight mass spectrometry
MIC	Minimum inhibitory concentration
LB	Luria–Bertani
TEM	Transmission electron microscopy
DLS	Dynamic light scattering
FTIR	Fourier transform infrared spectroscopy
XRD	X-ray diffraction
TGA	Thermogravimetric analysis
OD	Optical density
PBS	Phosphate-buffered saline
SEM	Scanning electron microscopy

## References

[1] Dini I., De Biasi M., Mancusi A. (2022). An overview of the potentialities of antimicrobial peptides derived from natural sources. Antibiotics.

[2] MacNair C.R., Rutherford S.T., Tan M.W. (2024). Alternative therapeutic strategies to treat antibiotic-resistant pathogens. Nat. Rev. Microbiol..

[3] Garvey M. (2023). Antimicrobial use in animal food production. Biodiversity, Functional Ecosystems and Sustainable Food Production.

[4] Quah Y., Tong S., Bojarska J., Giller K., Tan S.M., Ziora Z.M., Esatbeyoglu T., Chai T.T. (2023). Bioactive peptide discovery from edible insects for potential applications in human health and agriculture. Molecules.

[5] Sun S.W. (2024). Progress in the identification and design of novel antimicrobial peptides against pathogenic microorganisms. Probiotics Antimicrob. Proteins.

[6] Xuan J.Q., Feng W.G., Wang J.Y., Wang R.C., Zhang B.W., Bo L.T., Chen Z.S., Yang H., Sun L. (2023). Antimicrobial peptides for combating drug-resistant bacterial infections. Drug Resist. Updates.

[7] Moretta A., Scieuzo C., Petrone A.M., Salvia R., Manniello M.D., Franco A., Lucchetti D., Vassallo A., Vogel H., Sgambato A. (2021). Antimicrobial peptides: A new hope in biomedical and pharmaceutical fields. Front. Cell. Infect. Microbiol..

[8] Nesa J., Jana S.K., Sadat A., Biswas K., Kati A., Kaya O., Mondal R., Dam P., Thakur M., Kumar A. (2022). Antimicrobial potential of a ponericin-like peptide isolated from *Bombyx mori* L. hemolymph in response to *Pseudomonas aeruginosa* infection. Sci. Rep..

[9] Sultana A., Luo H., Ramakrishna S. (2021). Harvesting of antimicrobial peptides from insect (*Hermetia illucens*) and its applications in food packaging. Appl. Sci..

[10] Menk J.J., Matuhara Y.E., Sebestyen-França H., Henrique-Silva F., Ferro M., Rodrigues R.S., Santos-Júnior C.D. (2023). Antimicrobial peptide arsenal predicted from the venom gland transcriptome of the tropical trap-jaw ant *Odontomachus chelifer*. Toxins.

[11] Yang B., Yang H.Y., Liang J.L., Chen J.R., Wang C.H., Wang Y.Y., Wang J.C., Luo W.H., Deng T., Guo J.L. (2025). A review on the screening methods for the discovery of natural antimicrobial peptides. J. Pharm. Anal..

[12] Zhao Q., He L., Wang X.F., Ding X.S., Li L.G., Tian Y., Huang A.X. (2022). Characterization of a novel antimicrobial peptide isolated from *Moringa oleifera* seed protein hydrolysates and its membrane-damaging effects on *Staphylococcus aureus*. J. Agric. Food Chem..

[13] Tang Q., Tan P., Dai Z.L., Wang T., Xu S.R., Ding Y.K., Jin J.Q., Zhang X., Zhang Y.C., Zhou C.L. (2023). Hydrophobic modification improves the delivery of cell-penetrating peptides to eliminate intracellular pathogens in animals. Acta Biomater..

[14] Thitirungreangchai T., Roytrakul S., Aunpad R. (2024). Deciphering the intracellular action of the antimicrobial peptide A11 via an in-depth analysis of its effect on the global proteome of *Acinetobacter baumannii*. ACS Infect. Dis..

[15] Ahmad F., Salem-Bekhit M.M., Khan F., Alshehri S., Khan A., Ghoneim M.M., Wu H., Taha E.I., Elbagory I. (2022). Unique properties of surface-functionalized nanoparticles for bio-application: Functionalization mechanisms and importance in application. Nanomaterials.

[16] Nguyen M.D., Tran H., Xu S., Lee T.R. (2021). Fe3O4 nanoparticles: Structures, synthesis, magnetic properties, surface functionalization, and emerging applications. Appl. Sci..

[17] Popova V., Dmitrienko E., Chubarov A. (2023). Magnetic nanocomposites and imprinted polymers for biomedical applications of nucleic acids. Magnetochemistry.

[18] Mourdikoudis S., Kostopoulou A., LaGrow A.P. (2021). Magnetic nanoparticle composites: Synergistic effects and applications. Adv. Sci..

[19] Mishra N.N., Bayer A.S., Tran T.T., Shamoo Y., Mileykovskaya E., Dowhan W., Guan Z., Arias C.A. (2012). Daptomycin resistance in enterococci is associated with distinct alterations of cell membrane phospholipid content. PLoS ONE.

[20] Bhardwaj P., Hans A., Ruikar K., Guan Z., Palmer K.L. (2018). Reduced chlorhexidine and daptomycin susceptibility in vancomycin-resistant *Enterococcus faecium* after serial chlorhexidine exposure. Antimicrob. Agents Chemother..

[21] Čopič A., Dieudonné T., Lenoir G. (2023). Phosphatidylserine transport in cell life and death. Curr. Opin. Cell Biol..

[22] Criado-Navarro I., Mena-Bravo A., Calderón-Santiago M., Priego-Capote F. (2019). Determination of glycerophospholipids in vegetable edible oils: Proof of concept to discriminate olive oil categories. Food Chem..

[23] Ba-Abbad M.M., Benamor A., Ewis D., Mohammad A.W., Mahmoudi E. (2022). Synthesis of Fe_3_O_4_ nanoparticles with different shapes through a co-precipitation method and their application. JOM.

[24] Jannah R., Onggo D. (2019). Synthesis of Fe_3_O_4_ nanoparticles for color removal of printing ink solution. J. Phys. Conf. Ser..

[25] Vestergaard M.D.C., Nishida Y., Tran L.T.T., Sharma N., Zhang X., Nakamura M., Oussou-Azo A.F., Nakama T. (2023). Antifungal activity and molecular mechanisms of copper nanoforms against *Colletotrichum gloeosporioides*. Nanomaterials.

[26] Wang H., Luo J.C., Zhang Y.H., He D., Jiang R., Xie X.M., Yang Q., Li K.L., Xie J.X., Zhang J.Q. (2020). Phospholipid/hydroxypropyl-*β*-cyclodextrin supramolecular complexes are promising candidates for efficient oral delivery of curcuminoids. Int. J. Pharm..

[27] Zhao Y., Fan Q.L., Wang X.Y., Jiang X.X., Jiao L.Y., Liang W.Y. (2019). Application of Fe_3_O_4_ coated with modified plant polyphenol to harvest oleaginous microalgae. Algal Res..

[28] Wang Y., Wei Y.Y., Gao P.C., Sun S., Du Q., Wang Z.F., Jiang Y. (2021). Preparation of Fe_3_O_4_@PMAA@Ni microspheres towards the efficient and selective enrichment of histidine-rich proteins. ACS Appl. Mater. Interfaces.

[29] Peng S.J., Ouyang B.S., Men Y.Z., Du Y., Cao Y.B., Xie R.H., Pang Z.Q., Shen S., Yang W.L. (2020). Biodegradable zwitterionic polymer membrane coating endowing nanoparticles with ultra-long circulation and enhanced tumor photothermal therapy. Biomaterials.

[30] Çimen D., Bereli N., Denizli A. (2020). Metal-chelated magnetic nanoparticles for protein C purification. Sep. Sci. Technol..

[31] Rahmatika A.M., Toyoda Y., Nguyen T.T., Goi Y., Kitamura T., Morita Y., Kume K., Ogi T. (2020). Cellulose nanofiber and magnetic nanoparticles as building blocks constructing biomass-based porous structured particles and their protein adsorption performance. ACS Sustain. Chem. Eng..

[32] Poplewska I., Strachota B., Strachota A., Poplewski G., Antos D. (2024). Thermo- and pH-responsible gels for efficient protein adsorption and desorption. Molecules.

[33] Verma C., Verma D.K., Berdimurodov E., Barsoum I., Alfantazi A., Hussain C.M. (2024). Green magnetic nanoparticles: A comprehensive review of recent progress in biomedical and environmental applications. J. Mater. Sci..

[34] Murray C.J.L., Ikuta K.S., Sharara F., Swetschinski L., Aguilar G.R., Gray A., Han C., Bisignano C., Rao P., Wool E. (2022). Global burden of bacterial antimicrobial resistance in 2019: A systematic analysis. Lancet.

[35] Tang H.Z., Qiu L.F., He L., Zhao L., Qin R., Nan X.Y., Liu Z.Z., Bai P.L. (2025). Self-assembled stable magnetic COF super-particles through electrostatic interaction for efficient removal and detection of PFAS combined with LC-MS/MS. Adv. Funct. Mater..

[36] Lee J.H., Chung H., Shin Y.P., Kim I., Natarajan S., Veerappan K., Seo M., Park J., Hwang J.S. (2020). Transcriptome analysis of *Psacothea hilaris*: De novo assembly and antimicrobial peptide prediction. Insects.

[37] Ambli M., Deracinois B., Jenequin A., Ravallec R., Cudennec B., Flahaut C. (2023). Impact of bioinformatics search parameters for peptides’ identification and their post-translational modifications: A case study of proteolysed gelatines from beef, pork, and fish. Foods.

[38] Scieuzo C., Giglio F., Rinaldi R., Lekka M.E., Cozzolino F., Monaco V., Monti M., Salvia R., Falabella P. (2023). In vitro evaluation of the antibacterial activity of the peptide fractions extracted from the hemolymph of *Hermetia illucens* (Diptera: Stratiomyidae). Insects.

[39] Shin M.K., Park H., Hwang I., Bu K., Jang B., Lee S., Oh J.W., Yoo J.S., Sung J. (2023). In silico-based design of a hybrid peptide with antimicrobial activity against multidrug-resistant *Pseudomonas aeruginosa* using a spider toxin peptide. Toxins.

[40] Manniello M.D., Moretta A., Salvia R., Scieuzo C., Lucchetti D., Vogel H., Sgambato A., Falabella P. (2021). Insect antimicrobial peptides: Potential weapons to counteract antibiotic resistance. Cell. Mol. Life Sci..

[41] Baharin N.H.Z., Mokhtar N.F.K., Desa M.N.M., Dzaraly D., Muthanna A., Hashim A.M., Lani M.N., Shuhaimi M. (2023). Safety evaluation of secretome proteins from *Paenibacillus polymyxa* Kp10 and *Lactococcus lactis* Gh1 as the potential antimicrobial therapeutic agent. Halalsphere.

[42] Arunima S., Das A., Kalita P.J., Patil R.I., Pandey N., Bhattacharjee M., Das D., Acharjee S. (2024). Improved methods for total and chloroplast protein extraction from *Cajanus* species for two-dimensional gel electrophoresis and mass spectrometry. PLoS ONE.

[43] Sakoulas G., Bayer A.S., Pogliano J., Tsuji B.T., Yang S., Mishra N.N., Nizet V., Yeaman M.R., Moise P.A. (2012). Ampicillin enhances daptomycin- and cationic host defense peptide-mediated killing of ampicillin- and vancomycin-resistant *Enterococcus faecium*. Antimicrob. Agents Chemother..

[44] Jabbari Shiadeh S.M., Pormohammad A., Hashemi A., Lak P. (2019). Global prevalence of antibiotic resistance in blood-isolated *Enterococcus faecalis* and *Enterococcus faecium*: A systematic review and meta-analysis. Infect. Drug Resist..

[45] Zhang J.J., Li J.H., Peng Y.Z., Gao X.K., Song Q., Zhang H.Y., Elhag O., Cai M.M., Zheng L.Y., Yu Z.N. (2022). Structural and functional characterizations and heterologous expression of the antimicrobial peptides, Hidefensins, from black soldier fly, *Hermetia illucens* (L.). Protein Expr. Purif..

[46] Miao H.B., Wang L., Wu Q., Huang Z.X. (2024). Antimicrobial peptides: Mechanism, expressions, and optimization strategies. Probiotics Antimicrob. Proteins.

[47] Zubair H., She P.F., Li Y.M., Liu S.S., Li Z.H., Yang Y.F., Li L.H., Zhou L.Y., Wu Y. (2022). Study on antibacterial effect of halicin (SU3327) against *Enterococcus faecalis* and *Enterococcus faecium*. Pathog. Dis..

[48] Krishnan M., Choi J., Jang A., Kim Y. (2020). A novel peptide antibiotic, Pro10-1D, designed from insect defensin shows antibacterial and anti-inflammatory activities in sepsis models. Int. J. Mol. Sci..

[49] Squitieri D., Massaro F., Graziano M.M., Borocci S., Cacaci M., Di Vito M., Porcelli F., Rosato R., Ceccacci F., Sanguinetti M. (2024). Trematocine-derived antimicrobial peptides from the Antarctic fish *Trematomus bernacchii*: Potent antibacterial agents against ESKAPE pathogens. Front. Microbiol..

[50] Bertrams W., Lindhauer N.S., Rieke M.C., Paas A., Hoffmann K., Greene B., Visekruna A., Vilcinskas A., Seidel K., Schmeck B. (2021). *Tribolium castaneum* defensin 1 kills *Moraxella catarrhalis* in an in vitro infection model but does not harm commensal bacteria. Virulence.

[51] Asai M., Li Y., Khara J.S., Robertson B.D., Langford P.R., Newton S.M. (2019). *Galleria mellonella*: An infection model for screening compounds against the *Mycobacterium tuberculosis* complex. Front. Microbiol..

[52] Reichl B., Eichelberg N., Freytag M., Gojo J., Peyrl A., Buchberger W. (2020). Evaluation and optimization of common lipid extraction methods in cerebrospinal fluid samples. J. Chromatogr. B.

[53] Akhtar K., Javed K., Ali Shah S.S. (2023). Synthesis routes for multi-shape Fe_3_O_4_ nanoparticles through oxidation-precipitation of hematite and modified co-precipitation method without surfactant. J. Dispers. Sci. Technol..

[54] Kummari R., Puja R., Bose K. (2022). Protein quantitation and detection. Textbook on Cloning, Expression and Purification of Recombinant Proteins.

[55] Pimchan T., Hamzeh A., Siringgan P., Thumanu K., Hanboonsong Y., Yongsawadigul J. (2024). Antibacterial peptides from black soldier fly (*Hermetia illucens*) larvae: Mode of action and characterization. Sci. Rep..

[56] Zeng W.L., Han Y.J., Zheng X.K., Yao Z.C., Xu C.Q., Zhang X.T., Tang M.R., Shen M., Zhou T.L. (2023). Evaluation of resazurin microplate method for rapid detection of vancomycin and linezolid resistance in *Enterococcus faecalis* and *Enterococcus faecium* clinical isolates. J. Antimicrob. Chemother..

[57] Sim J.Y., Kim S., Lee J., Lim H., Kim H.H., Park Z.Y., Kim J.I. (2019). A significantly enhanced antibacterial spectrum of D-enantiomeric lipopeptide bactenecin. Biochem. Biophys. Res. Commun..

