# Supplementary material for: Phospholipid-Coated Fe3O4 Nanoparticles Enable Rapid Screening of Putative Antimicrobial Peptides from Clanis bilineata tsingtauica Hemolymph
Source: Antibiotics (Basel). 2026 Jul 18;15(7):702. doi: 10.3390/antibiotics15070702 (PMC13403957; doi:10.3390/antibiotics15070702)
Supplement: Supplementary file 1 [file antibiotics-15-00702-s001.zip › antibiotics-4426389-supplementary.pdf]

# Phospholipid-coated $Fe_3O_4$ nanoparticles enable rapid screening of putative antimicrobial peptides from *Clanis bilineata tsingtauica* hemolymph

Zong-Nan Li[1], Lei Qian[1], Qing-Yi Li[1, 2], Yi Qin[1], Pan Deng[1], Jian-Jun Guo[3], and Huai-Jian Liao[1, 2, *]

[1] *Institute of Leisure Agriculture, Jiangsu Academy of Agricultural Sciences, Nanjing 210014, China;*

[2] *College of Biotechnology, Jiangsu University of Science and Technology, Zhenjiang 212100, China;*

[3] *Provincial Key Laboratory for Agricultural Pest Management of Mountainous Region, Institute of Entomology, Guizhou University, Guiyang, Guizhou 550025, China;*

* Corresponding author. *E-mail*: lhj@jaas.ac.cn (Assoc. Prof. Dr. H. J. Liao).

**Tricine SDS PAGE**

The Tricine SDS-PAGE system (10 mL) was prepared using 9.5 mL of 12% separation gel and 2.5 mL of 4% stacking gel. The separation gel contained 3 mL of 30% acrylamide/bis-acrylamide (30:0.8, v/v), 2.5 mL of 1.5 M Tris-HCl (pH 8.8), 0.3 mL of 10% SDS, 3.2 mL of distilled water, 0.1 mL of 10% ammonium persulfate (APS), and 0.05 mL of TEMED. The stacking gel consisted of 0.5 mL of 30% acrylamide/bis-acrylamide, 1.25 mL of 0.5 M Tris-HCl (pH 6.8), 0.05 mL of 10% SDS, 0.6 mL of distilled water, 0.05 mL of 10% APS, and 0.025 mL of TEMED. During gel preparation, the separation gel was first pipetted into clamped glass plates, and anhydrous ethanol was layered on top to create a flat surface. After polymerization, the ethanol was removed, the stacking gel was added, and a 10-well comb was inserted and allowed to set. Each well was loaded with 5 µL of Color Mixed protein marker (11–180 kDa, Solarbio, PR1910, Beijing, China) or sample.

**Silver Staining**

The silver staining procedure was as follows: the fixing solution was prepared by mixing anhydrous ethanol, glacial acetic acid, and deionized water in a 5:1:4 (v/v/v) ratio, and the washing solution was prepared by mixing anhydrous ethanol and deionized water in a 1:4 (v/v) ratio. After electrophoresis, the gel was washed twice with deionized water for 5 minutes each. It was then incubated in 75 mL fixing solution on a shaker for 15 minutes, repeated once. The gel was rinsed in washing solution for 5 minutes, repeated once, and then treated with silver staining sensitizer for 2 minutes. After two 1-minute washes with deionized water, the gel was incubated with silver staining solution while shaking for 10 minutes, rinsed twice with deionized water for 30 seconds each at 60 rpm, then developed in silver staining developer at room temperature for 3 minutes. Staining was stopped by immediate addition of stop solution with agitation for 10 minutes. The gel was washed in 50 mL deionized water with shaking for 5 minutes, after which stained bands were excised and stored at 4 °C until analysis.

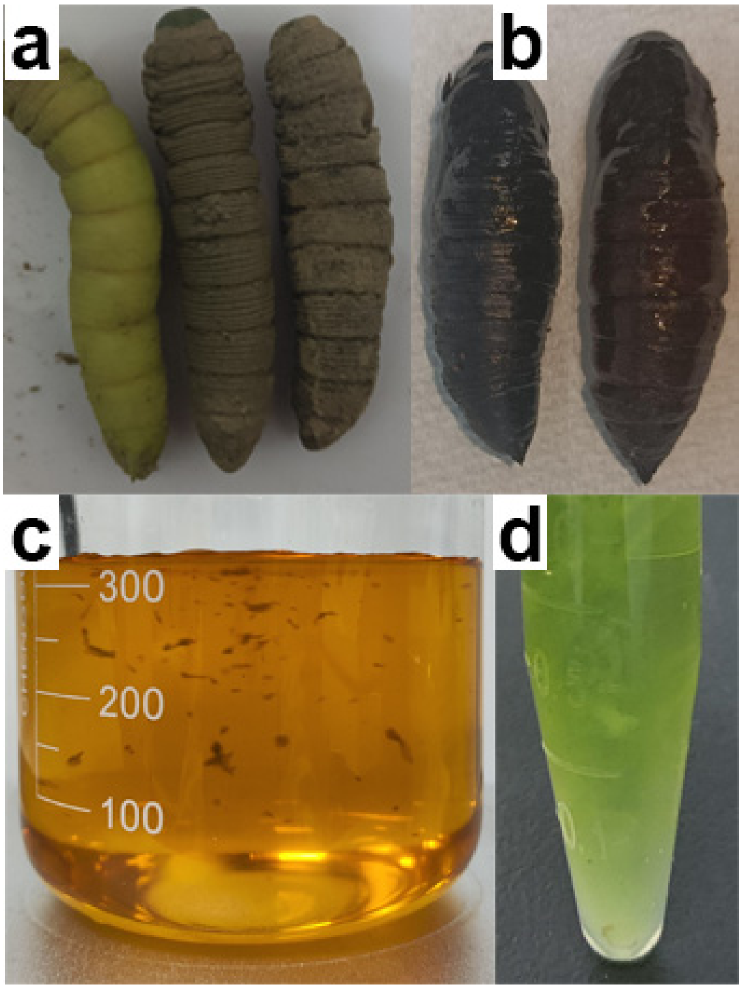


**Figure S1.** Experimental materials: (a) 5th instar larvae and (b) pupae of *C. bilineata tsingtauica*; (c) $Fe_3O_4$ nanoparticles synthesized by co-precipitation; (d) larval hemolymph; (e) $Fe_3O_4$@L nanoparticles with adsorbed putative AMPs. The leftmost larva was alive; the two on the right were dead and blackened. Both pupae were also dead, blackened, and decomposed.

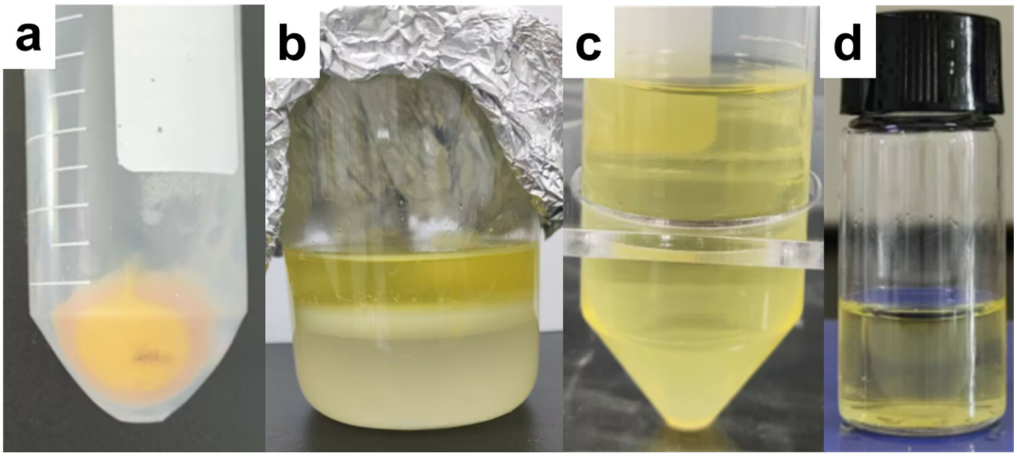


**Figure S2.** Materials for phospholipid extraction from *E. faecium* plasma membrane using the aqueous two-phase system: (a) *E. faecium* precipitate, (b) two-phase system, (c) organic phase containing phospholipids, (d) phospholipids re-dissolved in methanol.

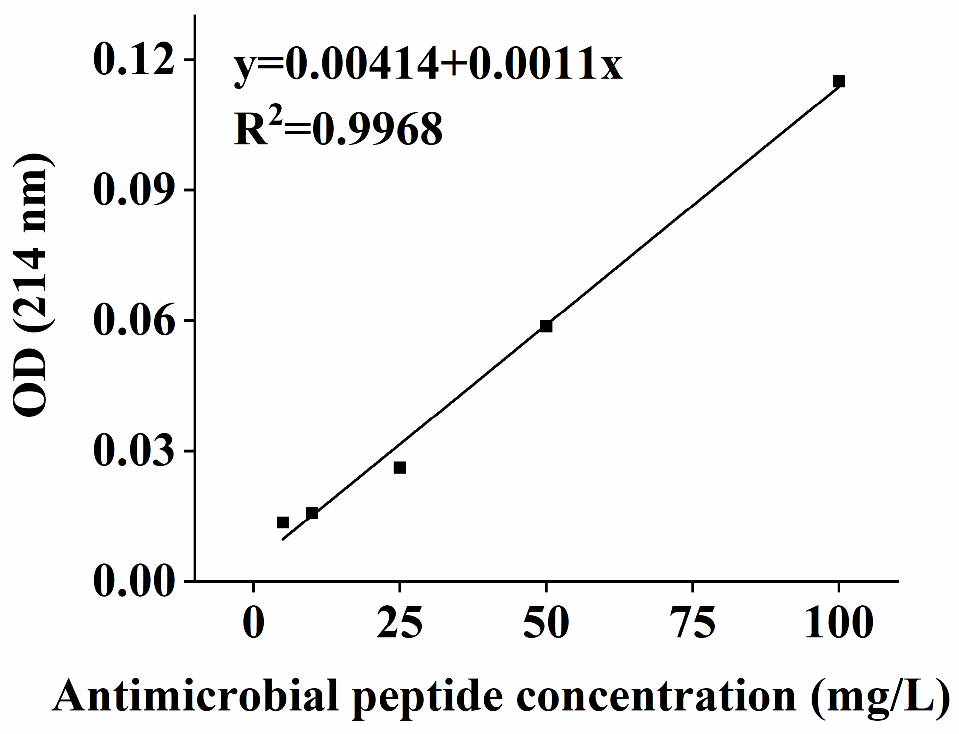


**Figure S3.** Standard curve for antimicrobial peptide concentration.

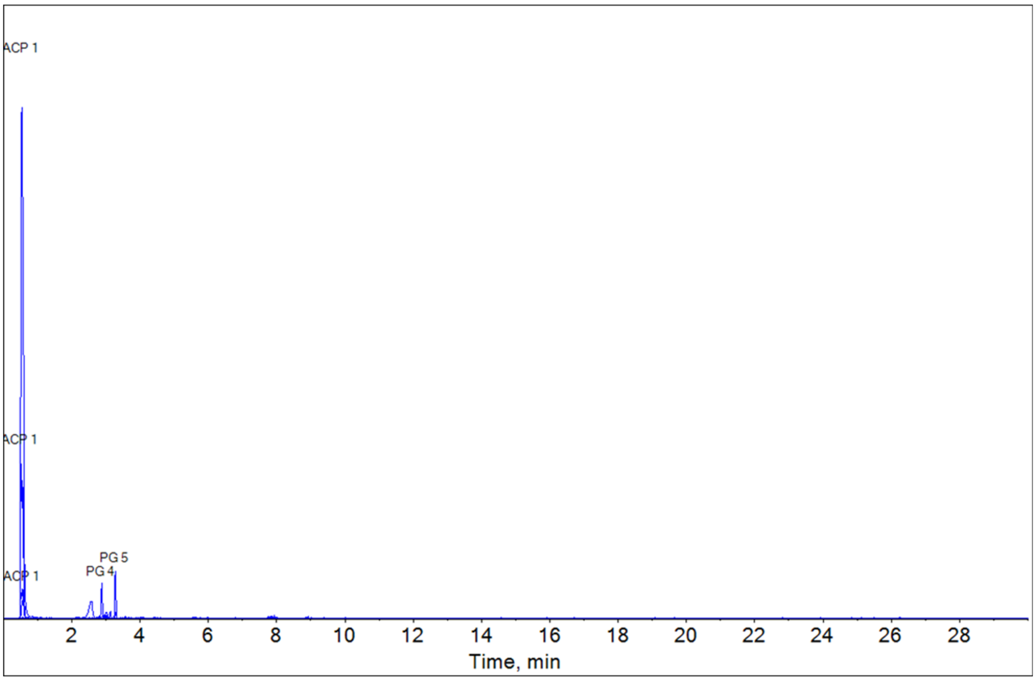


**Figure S4.** MALDI-TOF results of *E. faecium* membrane phospholipid analysis.

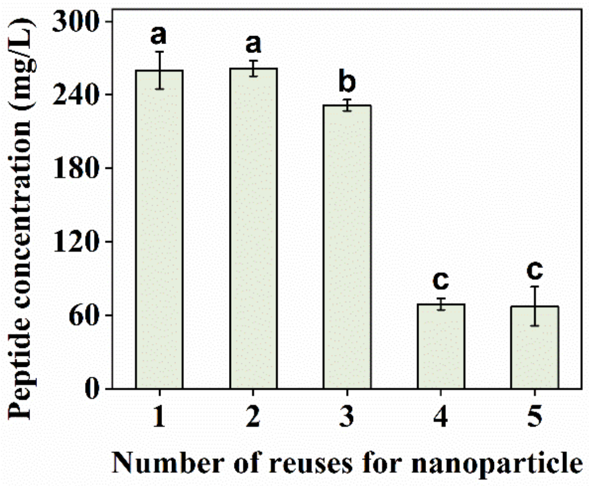


**Figure S5.** Effect of $Fe_3O_4$@L nanoparticle reuse on enrichment of hemolymph peptides from *C. bilineata tsingtauica* larvae. Different lowercase letters indicate significant differences ($p$ < 0.05).

**Table S1.** Phospholipid detection list.

| Abbreviation | Name | Molecular formula |
|---|---|---|
| ACP1 | amino-containing phospholipid 1 | $C_{42}H_{82}NO_{10}P$ |
| ACP2 | amino-containing phospholipid 2 | $C_{42}H_{78}NO_{10}P$ |
| ACP3 | amino-containing phospholipid 3 | $C_{34}H_{66}NO_{10}P$ |
| ACP4 | amino-containing phospholipid 4 | $C_{53}H_{86}N_9O_{12}P$ |
| ACP5 | amino-containing phospholipid 5 | $C_{28}H_{59}N_2O_6P$ |
| ACP6 | amino-containing phospholipid 6 | $C_{45}H_{93}N_2O_4PS$ |
| ACP7 | amino-containing phospholipid 7 | $C_{11}H_{16}N_3O_7PS$ |
| ACP8 | amino-containing phospholipid 8 | $C_{10}H_{16}N_3O_6PS$ |
| LPG1 | lysyl-phosphatidylylcerol 1 | $C_{40}H_{77}O_{10}P$ |
| LPG2 | lysyl-phosphatidylylcerol 2 | $C_{40}H_{75}O_{10}P$ |
| PG1 | phosphatidylglycerol 1 | $C_{40}H_{77}O_{10}P$ |
| PG2 | phosphatidylglycerol 2 | $C_{42}H_{75}O_{10}P$ |
| PG3 | phosphatidylglycerol 3 | $C_{34}H_{67}O_{10}P$ |
| PG4 | phosphatidylglycerol 4 | $C_{30}H_{59}O_{10}P$ |
| PG5 | phosphatidylglycerol 5 | $C_{38}H_{75}O_{10}P$ |
| PG6 | phosphatidylglycerol 6 | $C_{33}H_{64}NO_{11}P$ |
| PG7 | phosphatidylglycerol 7 | $C_3H_9O_6P$ |
| CL1 | cardiolipin 1 | $C_{13}H_{18}O_{17}P_2$ |
| CL2 | cardiolipin 2 | $C_{75}H_{142}O_{17}P_2$ |
| CL3 | cardiolipin 3 | $C_{73}H_{140}O_{17}P_2$ |
| GP-DGDAG1 | glycerolphospho-diglycodiacylglycerol 1 | $C_{39}H_{80}N_2O_{11}P_2$ |
| GP-DGDAG2 | glycerolphospho-diglycodiacylglycerol 2 | $C_{39}H_{80}N_2O_{11}P_2$ |
| PA1 | phosphatidic acid 1 | $C_{29}H_{57}O_{10}P$ |
| PA2 | phosphatidic acid 2 | $C_{39}H_{69}O_8P$ |

**Table S2.** TOF-MS results for *E. faecium* phospholipid samples.

| Compound Name | Formula | Adduct | Expected (m/z) | Foud at (m/z) |
|---|---|---|---|---|
| ACP1 | $C_{42}H_{82}NO_{10}P$ | $[M+H]^+$ | 792.5749 | 792.5847 |
| PG4 | $C_{30}H_{59}O_{10}P$ | $[M+H]^+$ | 611.3919 | 611.3960 |
| PG5 | $C_{38}H_{75}O_{10}P$ | $[M+NH_4]^+$ | 740.5436 | 740.5403 |
| PA2 | $C_{39}H_{69}O_8P$ | $[M+Na]^+$ | 719.4622 | 719.4615 |
